# Comparison of mesoporous fractal characteristics of silica-supported organocatalysts derived from bipyridine-proline and resultant effects on the catalytic asymmetric aldol performances†

**DOI:** 10.1039/d2ra00971d

**Published:** 2022-04-07

**Authors:** Guangpeng Xu, Liujie Bing, Bingying Jia, Shiyang Bai, Jihong Sun

**Affiliations:** Beijing Key Laboratory for Green Catalysis and Separation, Department of Environmental and Chemical Engineering, Beijing University of Technology Beijing 100124 China jhsun@bjut.edu.cn

## Abstract

Three kinds of the bipyridine-proline chiral ligands as highly active species were successfully introduced on Zn-modified mesoporous silica nanomaterials (BMMs, MCM-41, and SBA-15) *via* the covalent attachment and coordination methods. Their microstructural features and physicochemical properties were extensively characterized *via* XRD patterns, SEM/TEM images, TGA profiles, FT-IR and UV-Vis spectra. In particular, their fractal features, the pair distance distribution function, and the Porod plots were evaluated thoroughly on the basis of the SAXS data. Meanwhile, their catalytic performances for asymmetric aldol reactions between *p*-nitrobenzaldehyde and cyclohexanone were evaluated. The results indicated that the bimodal mesoporous BMMs-based samples with short worm-like mesoporous channels possessed both mass and surface fractal features, whereas the MCM-41- and SBA-15-based samples with long-range ordered structures only showed surface fractal features. The influences of various reaction parameters, including the textures of the mesoporous silicas, the structures of the used chiral ligands, and the molecular volumes of aldehydes, on the catalytic activities (yield) and stereoselectivities (dr and ee) were investigated thoroughly. The results showed satisfactory activities (yields) and better stereoselectivity (dr and ee) in comparison with the homogeneous catalytic system using Z as the catalysts. In particular, the 3^rd^ recycle catalytic performances of the Z-immobilized heterogeneous catalysts retained high catalytic yields (around 80%) and ee values of 28%. These phenomena were well interpreted by the essential relationships between the fractal characteristics of these heterogeneous catalysts and their catalytic activities.

## Abstract 2

The relationships between the fractal evolution and catalytic behavior of the resultant catalysts were deeply explored *via* SAXS method and other characterizations. The applicability and reliability of fractal theory are therefore proved.

## Graphical abstract

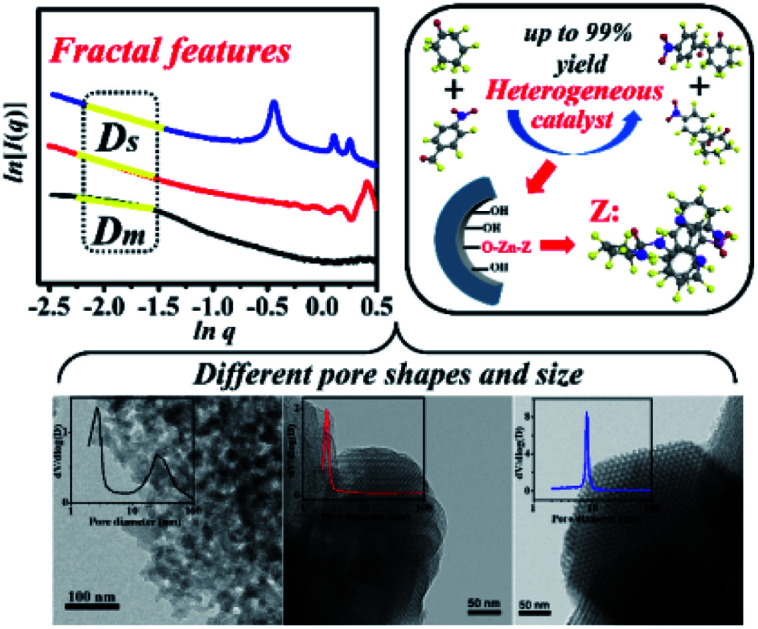

## 1. Introduction

In the last two decades, the asymmetric aldol reaction has developed into powerful strategies for constructing carbon–carbon bonds in an enantioselective fashion, and as an expansive area in synthetic organic chemistry.^1–3^ The design of efficient chiral ligands or related catalysts is also emerging as crucial components in exploratory asymmetric aldol catalysis research. Since List and Barbas (in 2000) first introduced proline into the asymmetric aldol reaction,^4,5^ proline-based derivatives consisting of two or more functional groups are extremely promising homogeneous catalysts as chiral building blocks and chiral ligands in modern asymmetric catalysis^6–9^ due to their low-cost, low toxicity, and environmental friendliness, as well as easy availability, mild reaction conditions, and high selectivity.^10,11^

Given these features, relevant explorations presently involve proline and their derivatives, such as amino acids,^12–14^ peptides,^8,15,16^ and chiral amides,^17^ as building blocks or ligands in the catalytic asymmetric reactions. A significant number of axially-unfixed 2,2′-bipyridine-based chiral bifunctional organocatalysts were synthesized using enantiopure l-proline as chiral sources. Their catalytic activity (yield) and stereoselectivity (dr and ee values) between cyclohexanone and *p*-nitrobenzaldehyde were assessed under the homogeneous aldol reaction, showing satisfactory catalytic performance.^18–21^

Despite their efficiency and selectivity, time-consuming and tedious procedures of separating the homogeneous catalysts are undeniable, especially for the complex reaction systems. Therefore, the utilization of heterogeneous catalysts is an excellent alternative to homogeneous catalysts.^22,23^ However, in order to provide useful catalytic materials for application in stereoselective transformations, how to maintain the activity and stereoselectivity of homogeneous catalysts is an important challenge for the heterogeneous immobilized-catalysts.

Ordered porous materials (MCM-41 or SBA-15) are employed as excellent catalyst supports owing to their abundant hydroxyl groups, extremely large surface area and controlled pore structure, as well as chemical inertness, and excellent thermal and mechanical stability in comparison with organic hosts.^24^ Based on these motivations, several heterogeneous proline-immobilized catalysts have recently been explored, while searching for an improvement in the catalytic efficiency and stereoselectivity.^25–27^

Our group (in 2003) reported early on that the bimodal mesoporous SiO_2_ (abbreviated as BMMs) consists of a small worm-like mesoporous structure in the size range of 2–3 nm and larger intra-particle mesopore distributions in the size range of 15–30 nm, showing a high surface area of around 700 m^2^ g^−1^ and large pore volume of up to 3.5 cm^3^ g^−1^.^28^ Comparably, MCM-41 or SBA-15 presents a highly oriented mesopore channel. Obviously, the modifiable surface and controllable bimodal mesopores would be more beneficial to the immobilization of the active species. Based on our previous studies, a variety of heterogeneous proline-immobilized catalysts were prepared by hydrogen bond or coordination bond method,^29–31^ showing higher catalytic activity for the asymmetric aldol reaction between *p*-nitrobenzaldehyde and cyclohexanone in comparison with that of the homogeneous catalysts.

**Scheme 1:**
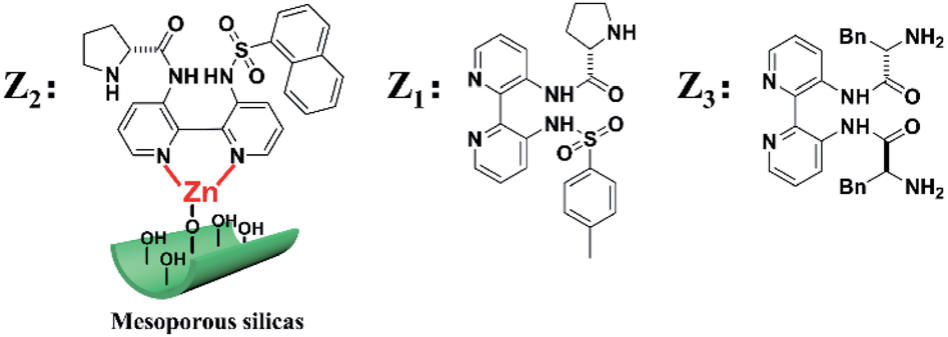
Three kinds of the Z moieties were introduced on Zn-modified mesoporous silica nanomaterials *via* covalent attachment and coordination methods.

Inspired by the above works, one of the primary goals of this study was to develop high-efficiency bipyridine-proline chiral heterogeneous catalysts. Herein, as shown in Scheme 1, the heterogeneous chiral bipyridine-proline catalysts based on the Zn-modified different supports (BMMs, MCM-41, and SBA-15) were prepared *via* a covalent attachment and coordination approach for exploration of the catalytic asymmetric aldol reactions. In this study, (*S*)-*N*-(3′-((4-methylphenyl)sulfonamido)-[2,2′-bipyridin]-3-yl)pyrrolidine-2-carboxamide and (2*S*,2′*S*)-*N*,*N*′-([2,2′-bipyridine]-3,3′-diyl)-bis(2-amino-3-phenylpropanamide) as highly active species were synthesized by our collaborator,^18–21^ and named as Z_1_ and Z_3_, respectively. Besides that, (*S*)-*N*-(3′-(naphthalene-1-sulfonamido)-[2,2′-bipyridin]-3-yl)-pyrrolidine-2-carboxamide was first synthesized by our group,^32^ and named as Z_2_, and its structural formula is shown in Scheme 1. Notably, these active species presented an excellent catalytic homogeneous aldol reaction.

However, during the preparation of the bipyridine-proline chiral heterogeneous catalysts, the dispersion of active components (Z_1_, Z_2_, and Z_3_) inevitably leads to the appearance of the fractal features. An in-depth exploration of these processes is very important for an essential understanding of the catalyst preparation process resulting from various controllable parameters. Some prior work in this area, the introduction of fractal features into the catalytic fields credited to Pfeifer and Avnir,^33,34^ brought valuable insights into heterogeneous catalysis. Indeed, the complex surfaces of the heterogeneous catalysts were thus treated as an object whose dimensions approximately ranged between 1–3, owing to many problems such as concave and convex features, broken folds, and defects on the surface of the heterogeneous catalyst, rather than based on the consideration of simple two-dimensional surfaces, so that the reactions on the catalytic surfaces could be represented by fractal analysis.^35^ Unfortunately, few methods are available to measure this irregularity of shapes in heterogeneous catalysis.^36^ Small-angle X-ray scattering (SAXS) has been a rapid and non-destructive detection method with high sensitivity in recent years, which can be utilized to obtain quick and accurate information (including the fractal dimension, the characteristic shape and size, and the thickness of the interfacial layer) about the microstructure of mesoporous materials.^37–46^ The SAXS method has been successfully applied for functionalizing mesoporous silicas by Li *et al.*^37–42^ and other investigators.^43,44^

More recently, our group demonstrated the effects of Z-immobilized amounts on the catalytic performances for the asymmetric aldol reaction on the basis of the fractal evolutions derived from the SAXS data.^47^ Therefore, this motivates us to further explore the relationship between the fractal structural features of the resultant catalysts and their catalytic behaviors in mesoporous silicas with different pore shapes and size.

In this work, the abovementioned heterogeneous catalysts were deeply investigated by the SAXS methods, and its usefulness was also checked for BMMs-, MCM-41-, and SBA-15-based heterogeneous catalysts. This provides a facile route to engineer heterogeneous catalysts with high catalytic performance.

Another key novelty is that the synthesized heterogeneous catalysts with different nanoporous structures showed high catalytic activity and better stereoselectivity in asymmetric aldol reactions of *p*-nitrobenzaldehyde with cyclohexanone, and even using bulkier aldehyde derivatives (2-naphthaldehyde, 9-anthracenecarboxaldehyde, and 1-pyrenecarboxaldehyde) as reactive substrates. Moreover, the effect mechanism of the mesopore size and morphology of the BMMs-, MCM-41-, and SBA-15-based heterogeneous catalysts were demonstrated so as to clarify the associations between the fractal features and their catalytic performances, including the catalytic activity (yields) and stereoselectivity (dr and ee), especially between the fresh and recycled catalysts. The essence of these relationships is that the active species (Z) entering or partially blocking the mesoporous channels had different dispersion states on the surface of the supports with various fractal characteristics.

Meanwhile, the microstructural features and physicochemical properties of all samples were extensively characterized using powder X-ray diffraction (XRD) patterns, N_2_ adsorption and desorption isotherms, thermogravimetric analysis (TGA) curves, inductively coupled plasma-optical emission spectroscopy (ICP-OES) and elemental analysis, UV-visible (UV-Vis) and Fourier transform infrared spectroscopy (FT-IR) spectra, scanning (and transmission) electron microscopy (SEM and TEM), and energy-dispersive X-ray (EDX) spectroscopy. The products derived from the asymmetric aldol reactions in aqueous media were determined by high-performance liquid chromatography (HPLC) analysis.

## 2. Experimental

### 2.1 Catalysts synthesis

The synthesis procedure of BMMs was partially based on the method reported by Sun *et al.*^28^ According to the literature reported by Kresge *et al.*,^48^ mesoporous MCM-41 was synthesized on the basis of the molar ratio of the starting composition, as follows: H_2_O : NH_4_OH : CTAB : TEOS = 139 : 5.14 : 0.14 : 1. In a typical procedure, 2.5 g of CATB was dissolved in 120 mL of deionized water. Then, 9.5 mL of NH_4_OH (25%) was added to the above solution, followed by 10.5 mL of TEOS. The solution was stirred vigorously at room temperature for 1 h, and the suspension was then transferred into 300 mL Teflon-lined stainless-steel autoclaves for hydrothermal reactions at 130 °C for 72 h. After cooling down to room temperature, the resulting precipitates were collected by Buchner funnel and the solid residue was washed repeatedly with deionized water until solution neutrality. The resulting precipitates were dried at 60 °C for 8 h in the vacuum oven and subsequently heated to 550 °C for 5 h at a rate of 2 °C min^−1^ in a muffle furnace under atmospheric air. Finally, the MCM-41 powder was obtained by grinding the calcined product. The synthesis of hexagonal silica SBA-15 was prepared using P123 as a template and TEOS as a silica source, according to the literature reported by Coppens *et al.*^28^ A typical gel composition in terms of molar ratio was TEOS : HCl : H_2_O : P123 = 1 : 5.88 : 204 : 0.017. A portion of 4.16 g of P123 was first dissolved with stirring in a mixture of 155 mL of deionized water and 20.8 mL of 2 M HCl at 35 °C and 9.5 mL of TEOS was then added. The resulting mixture solution was stirred at 35 °C for 24 h, and followed by hydrothermal treatment at 100 °C for 24 h. The resulting solid was recovered by filtration and dried at 60 °C for 8 h in the vacuum oven. The organic template was removed by calcination under atmospheric air at 550 °C for 6 h at a rate of 1 °C min^−1^. Finally, the SBA-15 powder was obtained by grinding the calcined product.

Prior to the reaction, 0.5 g of well-powdered mesoporous silicas (BMMs, or MCM-41, or SBA-15) were separately added to each of the round bottom flasks (100 mL), and subsequently vacuum activated in an oil bath at 120 °C for 3 h. Meanwhile, zinc acetate dihydrate as a modifier was dissolved in 50 mL of anhydrous methanol and then added to the above flasks, in which the molar ratio of Zn/Si was around 1 : 2. The resulting reaction was stirred for 10 h at room temperature and the solvent was removed rapidly under vacuum at 50 °C, and then dried in a vacuum oven at 60 °C for 8 h. Subsequently, the obtained residue was repeatedly washed by deionized water and anhydrous methanol, and finally dried at 60 °C for 8 h under vacuum. These modified mesoporous materials were denoted as ZnBMMs, ZnMCM-41, and ZnSBA-15, respectively.

A series of bipyridine-proline ligands (Z_1_, Z_2_, and Z_3_) were synthesized in accordance with a procedure previously published in the literature.^18–21,32^

A quantity of 0.3 g of Zn-modified mesoporous silicas (ZnBMMs, or ZnMCM-41, or ZnSBA-15) were separately added to each of the round bottom flasks (25 mL), which were vacuum activated in an oil bath at 80 °C for 3 h. After the activation process, the active compounds (Z_1_, or Z_2_, or Z_3_) were separately added to each of the flasks containing 2 mL of the CH_2_Cl_2_ solution (the molar ratios of Z_1_, Z_2_, and Z_3_ to Zn were each around 1 : 1). The resulting reaction was heated to 42 °C (CH_2_Cl_2_, boiling point 39.8 °C) in an oil bath and constantly stirred for 12 h under reflux. After the reaction, the solid in the reaction mixture was separated from the liquid phase by centrifugation, washed alternately by CH_2_Cl_2_ and DMSO, and dried at 60 °C for 8 h under vacuum. These resultant catalysts were sequentially named as Z_1_ (or Z_2_ or Z_3_)ZnBMMs, Z_1_(or Z_2_ or Z_3_)ZnMCM-41, and Z_1_(or Z_2_, or Z_3_)ZnSBA-15. For comparison, the same procedure was used as described above. The Z_2_-grafted BMMs were prepared without Zn by post-treatment and named Z_2_-BMMs.

### 2.2 Asymmetric aldol reaction

The reaction was tripled due to the cycle performance test. The specific procedure of the asymmetric aldol reaction was as follows: cyclohexanone (3.0 mmol, 315 μL) and distilled water (1.5 mL) were added to the reaction vial (10 mL) with TFA (20 mol%, 4.5 μL) and stirred at room temperature for 5 minutes. Then, the desired amounts of heterogeneous catalysts (Z_1_(or Z_2_, or Z_3_)ZnBMMs, Z_1_(or Z_2_, or Z_3_)ZnMCM-41, Z_1_(or Z_2_, or Z_3_)ZnSBA-15, corresponding to 20 mol% of each aromatic aldehyde) were added to the above solution and stirred for 5 minutes. Finally, the corresponding aromatic aldehyde (0.3 mmol) was added and the mixture was stirred for 120 h. All reactions were monitored for completion by thin-layer chromatography (TLC) using silica gel coated glass plates (60F254). After the reaction, the mixture solution was extracted with ethyl acetate (3 × 10 mL), and the combined organic extracts were dried with anhydrous Na_2_SO_4_ and concentrated in vacuo. Finally, the crude product was purified by column chromatography on silica gel (eluent: EtOAc/petroleum ether) to afford a mixture of *syn*- and *anti*-aldol products, and the dr and ee values were determined by chiral HPLC analysis.

### 2.3 Procedure for the recovery of the catalyst

The recycled catalyst (Z_2_ZnBMMs-100, Z_2_ZnMCM-41-100, and Z_2_ZnSBA-15-100) was separated from the reaction mixture by filtration and washed with the solvent (petroleum ether, 10 mL) three times. The collected catalyst was then dried under vacuum at 80 °C for 3 h, weighed, and reused for the next run, which was repeated three times.

## 3. Results and discussion

### 3.1 SAXS analysis

**Fig. 1:**
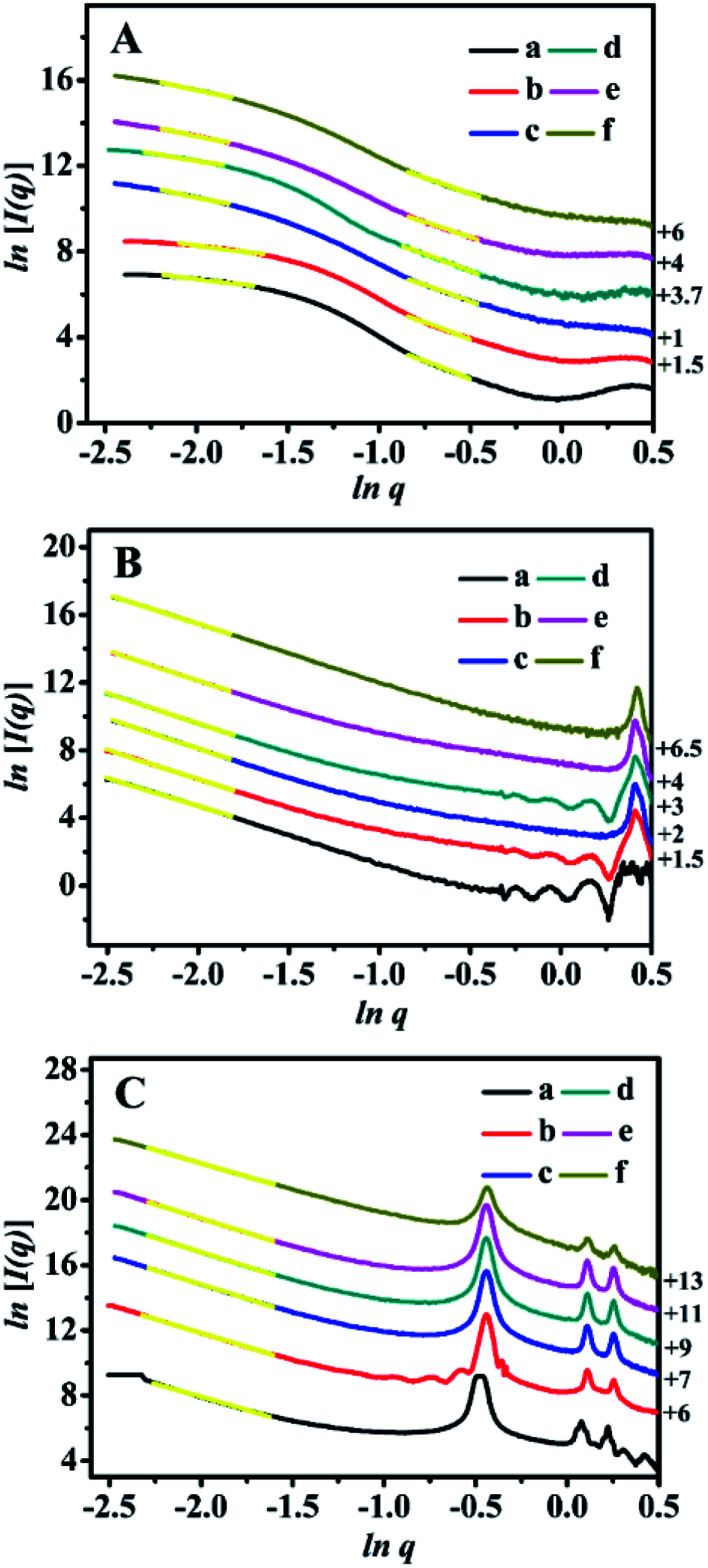
Shifted scattering curves (offset values in left *Y*-axis) of (A) BMMs-, (B) MCM-41-, (C) SBA-15-based samples, (a) pure mesoporous materials, (b) Zn-modified samples, (c) Z_1_-immobilized samples, (d) Z_2_-immobilized samples, (e) Z_3_-immobilized samples, and (f) 3^rd^ recycled Z_2_-immobilized samples. The molar ratios of Z/Zn for all samples were around 1 : 1.

**Fig. 2:**
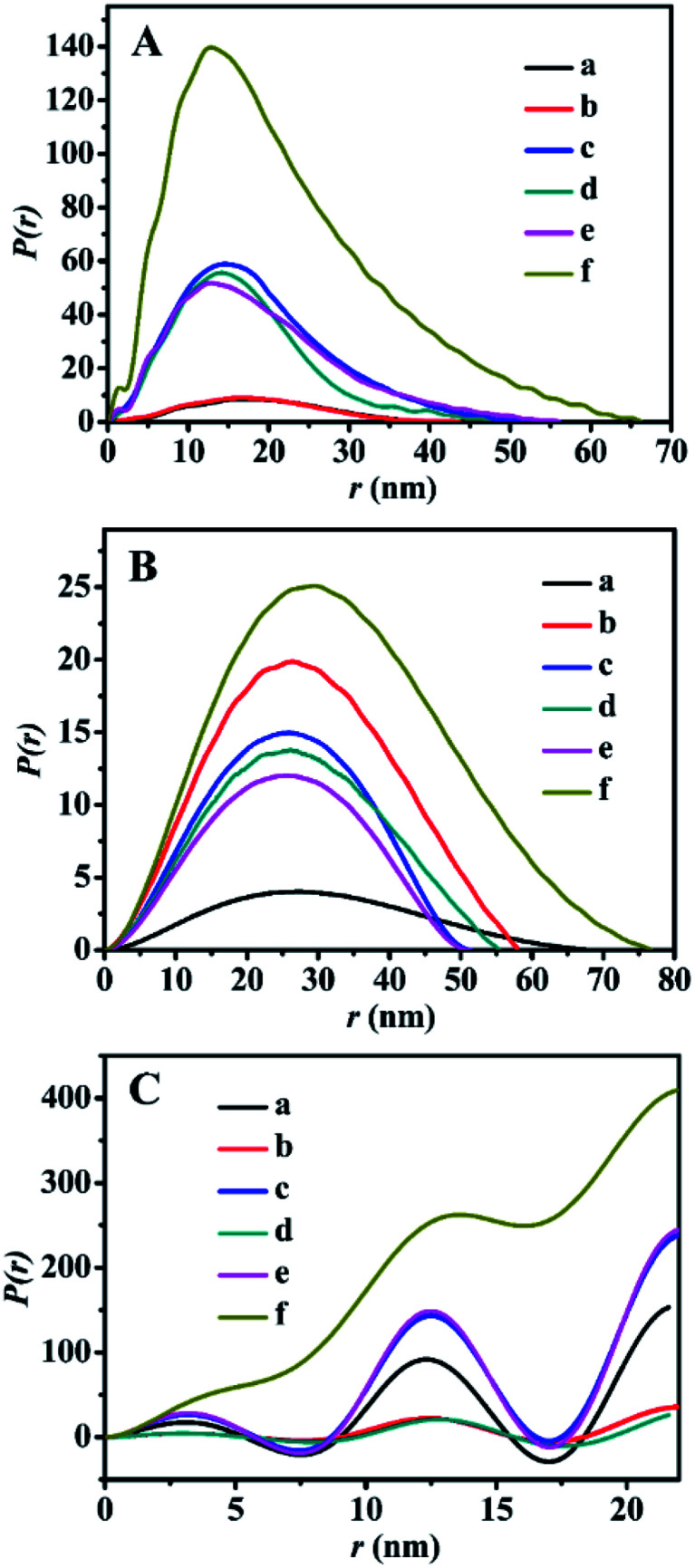
The PDDF profiles of (A) BMMs-, (B) MCM-41-, (C) SBA-15-based samples, (a) pure mesoporous materials, (b) Zn-modified samples, (c) Z_1_-immobilized samples, (d) Z_2_-immobilized samples, (e) Z_3_-immobilized samples, and (f) 3^rd^ recycled Z_2_-immobilized samples. The molar ratios of Z/Zn for all samples were around 1 : 1.

The SAXS patterns of the representative BMMs-, MCM-41-, and SBA-15-based samples are presented in Fig. 1. Their corresponding PDDF curves are shown in Fig. 2, and their related parameters are listed in Table 1.

**Table 1:** Collections of the fractal dimension values, linear range, possible maximum particle dimensions, Porod deviation, and average interface layer thickness values of various samples with different structures, and the possible relationships between the fractal dimension and their catalytic performance for the asymmetric aldol reaction between cyclohexanone and a variety of aromatic aldehydes^a^

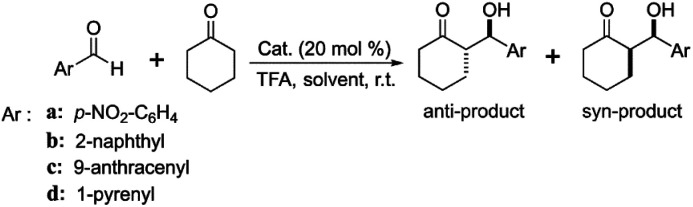												
Entry	Sample	Slope^b^		Fractal dimension^b^		Linear range (nm^−1^)	Maximum particle diameter^c^ (nm)	Porod deviation,^d^ average interface layer thickness values^e^ (nm)	Catalytic performance			
				Low-*q* region	High-*q* region				Reaction^f^	Yield^g^ (%)	dr^h^ (%)	ee^h^ (%)
1	BMMs	−1.01	−3.28	*D*_m_ = 1.01	*D*_s_ = 2.72	0.11 < *q* < 0.19	43	Positive, —	—	—	—	—
						0.43 < *q* < 0.61						
2	ZnBMMs	−1.10	−3.14	*D*_m_ = 1.10	*D*_s_ = 2.86	0.12 < *q* < 0.20	45	Negative, 1.61	—	—	—	—
						0.43 < *q* < 0.61						
3	Z_1_ZnBMMs-100	−1.79	−3.04	*D*_m_ = 1.79	*D*_s_ = 2.96	0.11 < *q* < 0.16	52	Negative, 0.45	a	97	73 : 27	39
									b	76	80 : 20	39
						0.43 < *q* < 0.63			c	19	91 : 9	3
									d	40	71 : 29	28
4	Z_2_ZnBMMs-100	−1.40	−3.13	*D*_m_ = 1.40	*D*_s_ = 2.87	0.10 < *q* < 0.16	47	Negative, 0.94	a	97	67 : 33	43
									b	84	77 : 23	36
						0.42 < *q* < 0.63			c	23	91 : 9	3
									d	38	70 : 30	31
5	Z_3_ZnBMMs-100	−1.78	−2.84	*D*_m_ = 1.78	*D*_m_ = 2.84	0.11 < *q* < 0.16	56	Negative, 1.44	a	57	59 : 41	53
									b	20	59 : 41	50
						0.43 < *q* < 0.63			c	11	94 : 6	8
									d	10	63 : 37	35
6	MCM-41	−3.28		*D*_s_ = 2.72		0.08 < *q* < 0.16	—	Positive, —	—	—	—	—
7	ZnMCM-41	−3.43		*D*_s_ = 2.57		0.08 < *q* < 0.16	—	Positive, —	—	—	—	—
8	Z_1_ZnMCM-41-100	−3.55		*D*_s_ = 2.45		0.08 < *q* < 0.16	—	Positive, —	a	99	73 : 27	28
									b	82	79 : 21	36
									c	27	83 : 17	6
									d	38	73 : 27	30
9	Z_2_ZnMCM-41-100	−3.54		*D*_s_ = 2.46		0.08 < *q* < 0.16	—	Positive, —	a	93	72 : 28	28
									b	70	80 : 20	38
									c	18	88 : 12	5
									d	28	74 : 26	36
10	Z_3_ZnMCM-41-100	−3.53		*D*_s_ = 2.47		0.08 < *q* < 0.16	—	Positive, —	a	77	57 : 43	35
									b	13	57 : 43	42
									c	16	79 : 21	9
									d	21	57 : 43	33
11	SBA-15	−3.21		*D*_s_ = 2.79		0.10 < *q* < 0.20	—	Positive, —	—	—	—	—
12	ZnSBA-15	−3.42		*D*_s_ = 2.58		0.10 < *q* < 0.20	—	Positive, —	—	—	—	—
13	Z_1_ZnSBA-15-100	−3.48		*D*_s_ = 2.52		0.10 < *q* < 0.20	—	Positive, —	a	94	76 : 24	36
									b	92	82 : 18	32
									c	23	82 : 18	2
									d	27	64 : 36	21
14	Z_2_ZnSBA-15-100	−3.49		*D*_s_ = 2.51		0.10 < *q* < 0.20	—	Positive, —	a	99	75 : 25	36
									b	96	78 : 22	35
									c	28	85 : 15	4
									d	43	73 : 27	27
15	Z_3_ZnSBA-15-100	−3.51		*D*_s_ = 2.49		0.10 < *q* < 0.20	—	Positive, —	a	53	57 : 43	38
									b	11	55 : 45	39
									c	11	79 : 21	5
									d	16	61 : 39	39
16	Z_2_ZnBMMs-100^i^	−1.82	−2.98	*D*_m_ = 1.82	*D*_m_ = 2.98	0.11 < *q* < 0.16	66	Negative, 0.95	a	85	58 : 42	28
						0.43 < *q* < 0.63						
17	Z_2_ZnMCM-41-100^i^	−3.41		*D*_s_ = 2.59		0.08 < *q* < 0.16	—	Positive, —	a	85	62 : 38	10
18	Z_2_ZnSBA-15-100^i^	−3.25		*D*_s_ = 2.75		0.10 < *q* < 0.20	—	Positive, —	a	81	60 : 40	18
19	Z_1_	—		—		—	—	—	a	94	71 : 29	24
20	Z_2_	—		—		—	—	—	a	97	72 : 28	28
21	Z_3_	—		—		—	—	—	a	55	50 : 50	29
22	Zn(OAc)_2_^j^	—		—		—	—	—	a	—	—	—
23	Z_1_-Zn(OAc)_2_^j^	—		—		—	—	—	a	92	74 : 26	32
24	Z_2_-Zn(OAc)_2_^j^	—		—		—	—	—	a	96	74 : 26	35
25	Z_3_-Zn(OAc)_2_^j^	—		—		—	—	—	a	58	62 : 38	39

a Reaction conditions: catalysts (20 mol% of substrate aldehyde, equivalent to 20 mol% of Z), aromatic aldehyde (0.3 mmol), cyclohexanone (3.0 mmol, 315 μL), distilled water (1.5 mL), and trifluoroacetic acid (TFA, 20 mol%), r.t., 120 h.

b Their following mass (or surface) fractal dimensions (*D*_m_ or *D*_s_) deriving from calculations on the basis of these scattering curves are generally determined by power-law decay.^42,47,49,50^ Slopes between −1 and −3 refer to the mass fractal structures (the mass fractal dimension, *D*_m_ = *α*, 1 < *D*_m_ < 3); slopes between −3 and −4 refer to the surface fractal (the surface fractal dimension *D*_s_ = 6 − *α*, 2 < *D*_s_ < 3).^47,49,50^

c Obtained from the PDDF analysis.

d Negative and positive deviation derived from Porod's law.

e The thickness values of an interfacial layer of the related samples were obtained by fitting the deviation directly, according to Porod's plots.

f Numbered reaction lower case letters indicate various substituted aromatic aldehyde substrates in the abovementioned aldol reaction. The molecular diameters of *p*-nitrobenzaldehyde, 2-naphthaldehyde, 9-anthracenecarboxaldehyde, and 1-pyrenecarboxaldehyde were measured by Chem3D software. Their corresponding values were found to be 0.69, 0.82, 0.96, and 0.97, respectively.

g Isolated yield after separation by silica gel.

h The diastereomeric and enantiomeric ratios were determined by chiral HPLC analysis.

i The recycled catalyst after three runs.

j A control experiment with the addition of Zn(OAc)_2_ was carried out.

As shown in Fig. 1A, the SAXS patterns of the BMMs-based samples show two-segment discontinuous linear characteristics in the measured *q* ranges. Because the slope values of Z_1_-, Z_2_-, and Z_3_ZnBMMs-100 (as shown in Table 1, entries 3–5) were between −1 and −3 in the low-*q* regions (0.10 < *q* < 0.20), their behaviors presented a *D*_m_ characteristic in values of 1.79, 1.40 and 1.78, respectively, which are larger than those of BMMs (1.01, as shown in Table 1, entry 1) and ZnBMMs (1.10, as shown in Table 1, entry 2). These results implied their greater densifications after Zn-modifications and subsequent Z-immobilizations. The possible reason is due to introducing a larger number of active species (Z_1_, Z_2_, and Z_3_) into the mesoporous surfaces of Zn-grafted BMMs for the successful coordination of nitrogen atoms on bipyridine with Si–O–Zn. Besides, the slope values between −3 and −4 (Table 1, entries 1–5) referred to the *D*_s_ features in the high-*q* region (0.42 < *q* < 0.63). The corresponding values were calculated to be in the range of 2.72–2.96 (except Z_3_ZnBMMs-100, as shown in Table 1, entry 5),^47,49,50^ indicating their densifications with rough surfaces (Table 1, entries 1–4). The possible reason may be related to the aggregation or dispersion of the active species on the inner and outer surfaces of BMMs, resulting in the coexistences of both *D*_m_ and *D*_s_.

As shown in Fig. 1B, the MCM-41-based samples possessed *D*_s_ characteristics in the linear range of the assay (0.08 < *q* < 0.16), in comparison with the behavior of BMMs-based samples. This indicated that the MCM-41-based particles possessed uniform dense structures with rough surfaces.

As shown in Table 1, their *D*_s_ values decreased from 2.72 for MCM-41 (Table 1, entry 6) to 2.57 for ZnMCM-41 (Table 1, entry 7), and continually declined to 2.45 for Z_1_ZnMCM-41-100 (Table 1, entry 8), 2.46 for Z_2_ZnMCM-41-100 (Table 1, entry 9), and 2.47 for Z_3_ZnMCM-41-100 (Table 1, entry 10). Obviously, these results implied that the rough surfaces gradually became smooth during the Zn-modification and subsequent Z-immobilization.

Similar results of the SBA-15-based samples were also observed in Fig. 1C. Their *D*_s_ values also presented the decreased tendencies from 2.79 (Table 1, entry 11) to 2.58 (Table 1, entry 12), and continually declined to 2.52 for Z_1_ZnSBA-15-100 (Table 1, entry 13), 2.51 for Z_2_ZnSBA-15-100 (Table 1, entry 14), and 2.49 for Z_3_ZnSBA-15-100 (Table 1, entry 15). Obviously, these results were a big difference from that of the BMM-based samples. One of the main reasons is due to Z-blocking in the long-ordered mesopores channels, which could be confirmed in the following demonstration of their pore size distributions.

Furthermore, their detailed morphological information (including the particle size and shape, as well as the interfacial structures between the immobilized-Z and modified-Zn) could be derived from the PDDF profiles.^45,47^ As shown in Fig. 2A, the PDDF curves of Z_1_(or Z_2_, or Z_3_)ZnBMMs (Fig. 2A-c, -d, and -e) with an approximately symmetric shape were similar to that of BMMs (Fig. 2A-a) and ZnBMMs (Fig. 2A-b). This suggested the appearances of globular particles, which can be further evidenced in the following demonstrations *via* SEM images. Notably, the maximum diameter (*D*_max_) of their particles could be estimated from where the PDDF decays to zero, which was around 52, 47, and 56 nm (Table 1, entries 3–5), respectively. These diameters are slightly larger than those of BMMs (43 nm, as shown in Table 1, entry 1) and ZnBMMs (45 nm, as shown in Table 1, entry 2). Based on the SAXS patterns of the BMMs-based samples, we could speculate that Z_1_(or Z_2_, or Z_3_)ZnBMMs with slightly bigger or similarly sized diameters of around 52, 47, and 56 nm (Table 1, entries 3–5) displayed a greater densification tendency with compact structures because of the appearances of the Si–O–Zn–Z interactions. These demonstrations indicate that a substantial portion of the Zn or Z species were successfully loaded into the inner-mesoporous surfaces of the BMMs.

Meanwhile, Fig. 2B shows the symmetric features of the PDDF profiles for ZnMCM-41 and Z_1_(or Z_2_, or Z_3_)ZnMCM-41-100, which are almost similar to that of pure MCM-41. These observations suggest the unobvious impacts of Zn-modification and Z-immobilization on their overall morphologies with the uniform oblate cylindrical structures.^25–27^ However, the PDDF patterns of the SBA-15-based samples (Fig. 2C) exhibited periodical fluctuations with an irregular sine wave shape. Comparably, the observed differences between the BMMs- and SBA-15-based samples may be related to their mesopore channel shapes, which can be further elucidated in the following discussion in the N_2_-sorption isotherms and TEM images.

As mentioned above, the values corresponding to the intersection of the curves with the *X*-axis in the PDDF diagrams probably refer to the maximum size of the particles, whereas the quantifiable range of the SAXS assay was determined to be several to tens of nanometers.^46^ Unfortunately, Fig. 2B and C show that the particle sizes (over 500 nm) of these MCM-41- or SBA-15-based samples were well beyond the range of the assay. Therefore, the aforementioned results determined based on the PDDF data may not be suitable to the present work.

Moreover, the two situations (negative and positive deviation) of the scattering characteristics for the BMMs-, MCM-41-, and SBA-15-based samples are illustrated in the ln[*q*^4^*I*(*q*)] ∼ *q*^2^ curves in Fig. S1 of the ESI section.† Herein, the existences of the interfacial layers between the immobilized-Z (or grafted-Zn) and mesoporous surfaces of BMMs can be roughly estimated by the negative deviation from Porod's law, and their structural parameters are summarized in Table 1.

As can be seen in Fig. S1-A, -F, and -K,† the scattering curves of the pure mesoporous materials (BMMs, MCM-41, and SBA-15) conformed well to the results of the positive deviation from Porod's law. In this regard, we could speculate that the existence of the unremoved template (CTAB) caused some additional scatterings.^37,38,47^ Comparably, the scattering curves of Z_1_(or Z_2_, or Z_3_)ZnBMMs-100 and ZnBMMs revealed a negative deviation from Porod's law (as shown in Fig. S1 from -B to -E†). The interfacial layer thickness between the immobilized-Z (or grafted Zn) and mesoporous surfaces of BMMs was around 0.45, 0.94, 1.44, and 1.61 nm (as shown in Table 1, entries 2–5).

Obviously, these demonstrations indicate that the Zn-modification and subsequent Z-immobilization actually caused their notable structural differences, besides their fractal dimensions (as shown in Fig. 1, 2, and S1†). Meanwhile, we also noted that the appearances of some additional scatterings stemming from the MCM-41- and SBA-15-based samples ultimately resulted in a positive deviation from Porod's law (as shown in Fig. S1 from -F to -O†).

The possible relationships between the abovementioned fractal features and the catalytic performance of these heterogeneous catalysts are discussed in the following section.

### 3.2 Catalytic performance for the asymmetric aldol reaction

In order to evaluate the influences of the above heterogeneous catalysts with different fractal structures on the catalytic performances for the asymmetric aldol reaction, the catalytic activity (yield) and stereoselectivity (dr and ee values) of various aromatic aldehydes (including *p*-nitrobenzaldehyde, 2-naphthaldehyde, 9-anthracenecarboxaldehyde, and 1-pyrenecarboxaldehyde) and cyclohexanone were investigated, and the obtained results are summarized in Table 1.

As can be seen in Table 1, the reaction performances of *p*-nitrobenzaldehyde and cyclohexanone were illustrated as examples. The catalytic activities of the resultant Z_1_(or Z_2_, or Z_3_)ZnBMMs-100 catalysts revealed the declined tendencies in yield and dr values with the increased molecular diameter from 1.19 nm (Z_2_) to 1.29 nm (Z_1_) and 1.34 nm (Z_3_). In detail, the corresponding yield and dr values were around 97% and 73 : 27 for Z_1_ZnBMMs-100 (as shown in Table 1, entry 3a), 97% and 67 : 33 for Z_2_ZnBMMs-100 (Table 1, entry 4a), 57% and 59 : 41 for Z_3_ZnBMMs-100 (Table 1, entry 5a), respectively. Herein, we tried to find possible associations between the fractal features of these heterogeneous catalysts and their asymmetric aldol catalytic performances.

As shown in Table 1, entries 3, 4, and 5, Z_1_- and Z_2_ZnBMMs-100 exhibited similar *D*_s_ values (2.96 and 2.87) with the higher surface irregularity, while Z_3_ZnBMMs-100 had distinctly *D*_m_ characteristics with the *D*_s_ value of 2.84. Obviously, this relationship might be relevant not only to the molecular sizes of active species (Z), but also to their dispersion behaviors on the Zn-modified surfaces of the BMMs matrix. In particular, the relationship would be relevant to the bimodal mesopore structures of the BMMs (entries 1–5 in Table 2) with the narrow small mesopores (around 2–3 nm) and broader larger mesopores (around 20–30 nm).^28–31,47^ Therefore, the resultant Z_1_- and Z_2_ZnBMMs-100 were selected as relatively efficient catalysts on the basis of the fractal structural demonstrations.

**Table 2:** Summaries of the structural properties and textural parameters of all samples

Entry	Sample^a^	BET surface area (m^2^ g^−1^)	Pore volume^b^ (cm^3^ g^−1^)	Small mean pore^c^ (nm)	Large mean pore^c^ (nm)
1	BMMs	1205	1.6	2.9	28.2
2	ZnBMMs	942	1.4	2.8	26.5
3	Z_1_ZnBMMs-100	632	0.8	2.7	25.0
4	Z_2_ZnBMMs-100	472	0.7	2.6	25.0
5	Z_3_ZnBMMs-100	750	0.9	2.6	26.7
6	Z_2_ZnBMMs-100^d^	470	0.7	2.7	27.4
7	MCM-41	710	0.7	2.9	—
8	ZnMCM-41	631	0.6	2.8	—
9	Z_1_ZnMCM-41-100	480	0.5	2.5	—
10	Z_2_ZnMCM-41-100	371	0.3	2.4	—
11	Z_3_ZnMCM-41-100	566	0.5	2.6	—
12	Z_2_ZnMCM-41-100^d^	464	0.5	2.8	—
13	SBA-15	635	1.1	9.2	—
14	ZnSBA-15	472	1.1	8.4	—
15	Z_1_ZnSBA-15-100	417	0.8	8.0	—
16	Z_2_ZnSBA-15-100	354	0.7	8.0	—
17	Z_3_ZnSBA-15-100	381	0.7	7.8	—
18	Z_2_ZnSBA-15-100^d^	379	0.9	8.9	—

a The molar ratio between the added amount of Z_1_, Z_2_, or Z_3_ to Zn was around 1 : 1.

b Estimated from the amounts adsorbed at a relative pressure (*P*/*P*_0_) of 0.99.

c The pore size distribution was calculated from the N_2_ desorption branches using the BJH method.

d The recycled catalyst after three runs.

Comparably, for the resultant Z_1_(or Z_2_, or Z_3_)ZnMCM-41-100, the corresponding *D*_s_ values were around 2.45, 2.46, and 2.47 (as shown in Table 1, entries 8–10), respectively. Interestingly, we found that their catalytic performances with *p*-nitrobenzaldehyde presented a small difference between the MCM-41- (Table 1, entries 8–10) and BMMs-based heterogeneous catalysts (Table 1, entries 3–5), whereas the *D*_s_ values were maintained around steady-state, and was slightly smaller than that of the BMMs-based catalysts. These results may be related to the existence of a large number of single small-ordered mesopores (Table 2, entries 7–11), which is similar to the narrow small mesopores of BMMs.^28–31^ Meanwhile, the *D*_s_ values of Z_1_(or Z_2_, or Z_3_)ZnSBA-15-100 were around 2.52, 2.51, and 2.49 (Table 1, entries 13–15), respectively, similar to that of the MCM-41-based catalysts. Their catalytic performances were also similar to those of the BMMs- and MCM-41-based catalysts. This could be attributed to the existences of a large number of single larger-ordered mesopores (Table 2, entries 13–17). In contrast, they were roughly three times larger than that of the MCM-41-based catalysts, and similar to the broader larger mesopores of BMMs.^28–31^

In summary, three types of silica-based catalysts exhibited similar or superior catalytic yields and stereoselectivity, as compared to that of Z_1_ (Table 1, entry 19), Z_2_ (Table 1, entry 20), Z_3_ (Table 1, entry 21), and Z_1_-, or Z_2_-, or Z_3_-Zn(OAc)_2_ (Table 1, entries 23–25). However, their catalytic activities are closely related to the molecular sizes of the used active species (Z). Therefore, the fractal characteristics of these heterogeneous catalysts may also be an important factor affecting the catalytic performances.

In particular, the bulkier aldehyde derivatives (2-naphthaldehyde, 9-anthracenecarboxaldehyde, and 1-pyrenecarboxaldehyde) used as reactants for asymmetric aldol reactions are also discussed. As can be seen in Table 1, the influences of the bipyridine-proline chiral structures and the molecular volume of aldehydes on the catalytic activity and stereoselectivity of the asymmetric aldol reaction were obvious in the presence of Z_1_(or Z_2_, or Z_3_)ZnBMMs-100, Z_1_(or Z_2_, or Z_3_)ZnMCM-41-100, and Z_1_(or Z_2_, or Z_3_)ZnSBA-15-100.

According to our previously reported procedure,^32,47^ the dynamic sizes of the Z_1_, Z_2_, and Z_3_ molecules were around 1.29, 1.19, and 1.34 nm, respectively. Obviously, their structural distinction is derived from the phenyl-methyl groups in Z_1_, naphthyl groups in Z_2_, and bulkier benzyl groups in Z_3_.

As shown in Table 1, the catalytic yields were very dependent on the bipyridine-proline structures of the used catalysts, especially in Z_3_ZnBMMs-100, which bore bulky benzyl groups or 1-pyrenyl groups (Table 1, entry 5). Its corresponding maximum value was only 57% for the aldol reaction between *p*-nitrobenzaldehyde and cyclohexanone (Table 1, entry 5), which is lower than 97% (entries 3 and 4 in Table 1) when using Z_1_ZnBMMs and Z_2_ZnBMMs as a catalyst. Obviously, the steric hindrance effect of the used catalysts is one of the key factors.^32,47^ Meanwhile, the catalytic activity decreased with the increased aldehyde molecular volume. For example, the obtained yield under the catalysis of Z_3_ZnBMMs decreased from 57 to 20% (entries 5a and b in Table 1) using 2-naphthaldehyde as a substrate, and 11, 10% (entries 5c and d in Table 1) when using 9-anthracenecarboxaldehyde and 1-pyrenecarboxaldehyde as a substrate, respectively. The possible reason for the low yields may be due to the enlarged molecular volume of the used reactants, which was equally associated with the steric hindrance effects.

On the other hand, the asymmetric aldol reaction of 9-anthracenecarboxaldehyde and cyclohexanone catalyzed by Z_1_(or Z_2_, or Z_3_)ZnBMMs-100 exhibited excellent diastereoselectivity (dr up to 91 : 9, 91 : 9, and 94 : 6, respectively) in aqueous media, whereas the ee value was relatively low (3, 3, and 8%, as shown in entries 3c, 4c, and 5c in Table 1). These phenomena may be attributed to the structural symmetry of 9-anthracenecarboxaldehyde reactant, as compared with others. In other words, the barriers for the active species attacking from the opposite side are symmetric when the aldehydes are activated, easily resulting in affording good diastereoselectivity. As can be seen in Table 1 (entries 3–5: a, b, and d), the dr values for the other reactions significantly decreased to 59 : 41, 59 : 41, and 63 : 37 with increasing complexity of the catalyst structure, respectively.

Table 1 summarizes the aldol reaction results between a variety of aromatic aldehydes and cyclohexanone in the presence of Z_1_(or Z_2_, or Z_3_)ZnMCM-41-100 and Z_1_(or Z_2_, or Z_3_)ZnSBA-15-100 in aqueous media. As listed in Table 1, entries 8–10, using *p*-nitrobenzaldehyde as a substrate under the catalysis of Z_1_ZnMCM-41-100, excellent catalytic activities for the desired aldol products were achieved, such as around 99% yield, higher dr value (73 : 27), and moderate ee value (28%). Meanwhile, under the catalysis of Z_2_ZnMCM-41-100 or Z_3_ZnMCM-41-100, the obtained yields decreased to 93% and 77%, respectively, and the dr values were around 72 : 28 and 57 : 43, respectively. As listed in Table 1 (entry 9), in the case of Z_2_ZnMCM-41-100 when using *p*-nitrobenzaldehyde, 2-naphthaldehyde, 9-anthracenecarboxaldehyde, and 1-pyrenecarboxaldehyde as the substrates, the yields were around 93%, 70%, 18%, and 28%, respectively. These results again suggest that the structure of the bipyridine-based chiral catalysts or the molecular size of the aromatic aldehydes have significant effects on the catalytic performances. As expected, similar results for Z_3_ZnMCM-41-100 (Table 1, entries 10b, c, and d) and Z_3_ZnSBA-15-100 (Table 1, entries 15b, c, and d) were also observed.

In particular, the reusability of representative heterogeneous catalysts (Z_2_ZnBMMs-100, Z_2_ZnMCM-41-100, and Z_2_ZnSBA-15-100) was also evaluated to assess the possibility for catalytic aldol reaction. As can be seen in Table 1, its 3^rd^ recycled catalytic activity showed 85% yield, 58 : 42 dr, and 28% ee for Z_2_ZnBMMs-100 (as shown in Table 1, entry 16), 85% yield, 62 : 38 dr, and 10% ee for Z_2_ZnMCM-41-100 (Table 1, entry 17), 81% yield, 60 : 40 dr, and 18% ee for Z_2_ZnSBA-15-100 (Table 1, entry 18). Obviously, these recycled heterogeneous catalysts can be reused, showing high yield and excellent diastereoselectivity.

Notably, to further clarify the applicability and reliability of the SAXS data analysis in this work, the possible association between the fractal structural and other important parameters needs to be established. In order to more deeply verify the successful incorporation of Z_1_, Z_2_, and Z_3_ on the mesoporous surface, all samples were characterized with XRD patterns, N_2_ sorption isotherms, SEM/TEM images, EDX spectroscopy, TGA profiles, ICP-OES and elemental analysis, FT-IR and UV-Vis spectra.

### 3.3 XRD patterns

**Fig. 3:**
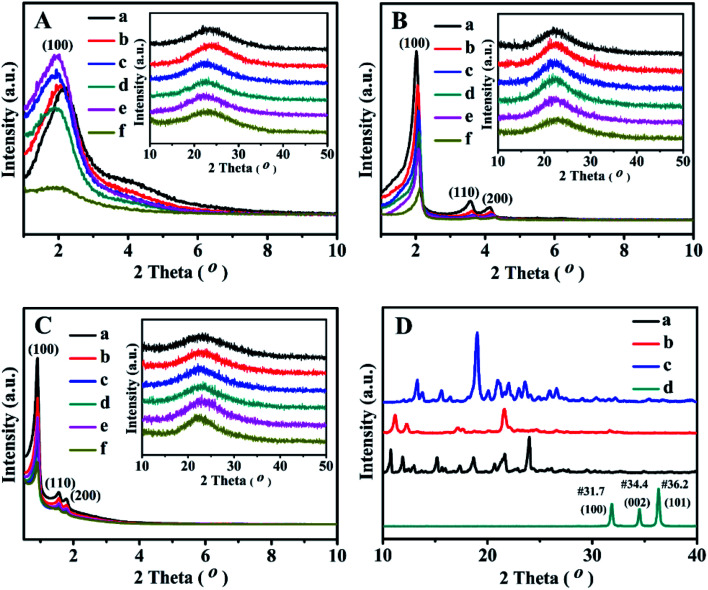
Small and wide (inset) angle XRD patterns of (A) BMMs-, (B) MCM-41-, and (C) SBA-15-based samples. (a) Pure mesoporous materials, (b) Zn-modified samples, (c) Z_1_-immobilized samples, (d) Z_2_-immobilized samples, (e) Z_3_-immobilized samples, and (f) 3^rd^ recycled Z_2_-immobilized samples, in which the molar ratios of Z/Zn for all samples were around 1 : 1. (D) Wide-angle XRD patterns of synthesized (a) Z_1_, (b) Z_2_, (c) Z_3_, and (d) ZnO.

Fig. 3 displays the XRD patterns of all related samples in the 2*θ* range of 1–10° and 10–50°. As shown in Fig. 3A, the single and relative broad diffractive (100) peaks of the as-prepared BMMs-based catalysts can be clearly identified at 2*θ* of around 2°, indicating the uniformly mesoporous structure of the typical BMMs.^28–30,47^

These results suggest that the mesopore structures of BMMs could remain intact after Zn-grafting (Fig. 3A-b) and Z immobilization (Fig. 3A-c, -d and -e). However, the peak_(100)_ position of ZnBMMs (Fig. 3A-b) moved slightly toward a lower angle region and its intensity was only slightly enhanced compared to that of BMMs (Fig. 3A-a). On the other hand, after immobilization with Z_1_, Z_2_, and Z_3_, respectively, the intensities of diffractive peak_(100)_ of Z_1_ZnBMMs-100 (Fig. 3A-c), Z_2_ZnBMMs-100 (Fig. 3A-d), and Z_3_ZnBMMs-100 (Fig. 3A-e) were higher than that of ZnBMMs (Fig. 3A-b). Their position was also shifted to the lower angle regions. These observations further demonstrated the successful immobilizations of the active species (Z_1_, Z_2_, and Z_3_) onto the mesoporous surface of the Zn-modified BMMs. On the basis of the above fractal evolution (Fig. 1) and PDDF analysis (Fig. 2), the enlarged crystal plane spacing (*d*) of the Zn-grafted BMMs and subsequent Z-immobilized BMMs further confirmed the formation of the interfacial layer thickness.^29,30,47^ Additionally, the introduction of the Zn or Z species could improve their ordered mesopore arrangements, which may be associated with the synthesis method of the used BMMs under a mild condition.^28,47^

As can be seen in Fig. 3B, the XRD patterns of the MCM-41-based samples showed three typical diffractive peaks at 2.02°, 3.57°, and 4.12°, corresponding to the diffractive indices of the (100), (110), and (200) planes of the order hexagonal lattice structures (Fig. 3B-a). Besides that, the XRD patterns of ZnMCM-41 (Fig. 3B-b) and Z_1_-, Z_2_-, and Z_3_ZnMCM-41-100 (Fig. 3B-c, -d, and -e) still retained these typical peaks, although their intensities were slightly weaker as compared with that of MCM-41 (Fig. 3B-a). Obviously, these observations indicated that the Zn-modification and Z-immobilization do not provoke significant destruction of the MCM-41 structures.

The XRD patterns of the synthesized SBA-15 (Fig. 3C-a) showed three well-resolved peaks at 0.91°, 1.55°, and 1.78°, which were assigned to the reflections of the (100), (110), and (200) planes of the 2D-hexagonal *P*6*mm* mesoporous structures.^51,52^ In addition, the Zn-modified (Fig. 3C-b) and Z_1_(or Z_2_, or Z_3_)-immobilized (Fig. 3C-c, -d, and -e) samples presented similar XRD patterns. However, the decrease in the intensity of these characteristic peaks for ZnSBA-15 (Fig. 3C-b) and Z_1_-, Z_2_-, or Z_3_ZnSBA-15 (Fig. 3C-c, -d and -e) signified the decline in the ordered mesopore regularity after Zn-modification or Z-immobilization.

Comparably, Fig. 3D shows the wide-angle XRD patterns of the synthesized active species (Z) and ZnO. As expected, there was no characteristic diffraction peak observed for ZnO and the Z series in Fig. 3A–C. Obviously, these results can be interpreted on the basis of covalent interactions between zinc acetate uniformly dispersed on the mesoporous surface and silanol groups, being consistent with a similar conclusion reported in the literature.^47,53^

### 3.4 N_2_ sorption isotherms

**Fig. 4:**
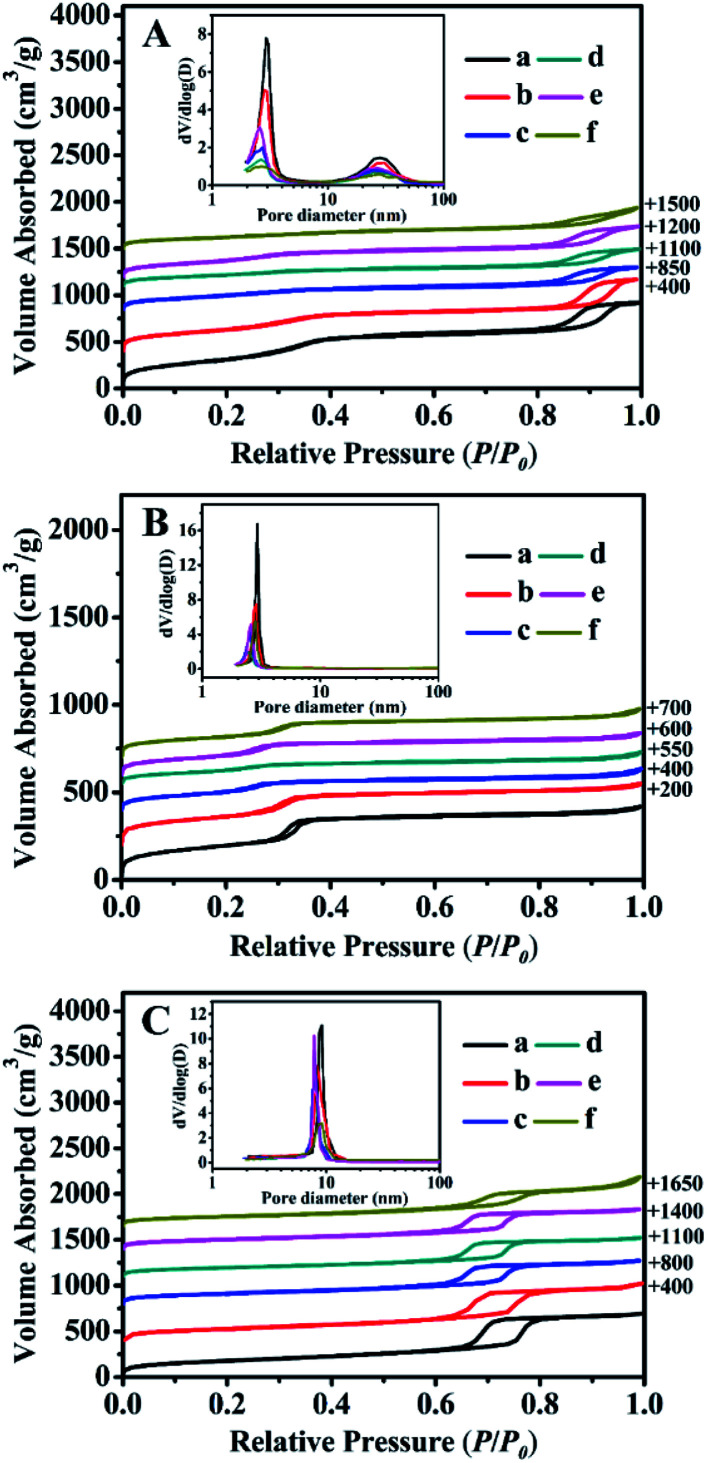
N_2_ adsorption–desorption isotherms and corresponding pore size distribution (inset) of (A) BMMs-, (B) MCM-41-, and (C) SBA-15-based samples. (a) Pure mesoporous materials, (b) Zn-modified samples, (c) Z_1_-immobilized samples, (d) Z_2_-immobilized samples, and (e) Z_3_-immobilized samples, and (f) 3^rd^ recycled Z_2_-immobilized samples, in which the molar ratios of Z/Zn for all samples were around 1 : 1.

The N_2_ adsorption–desorption isotherms and their corresponding pore size distributions (inset) of all samples are shown in Fig. 4. Their structural features and textural parameters are summarized in Table 2.

As can be seen in Fig. 4A, all of the isotherms were identified as type IV isotherms with H1 hysteresis loops, similar to a typical feature of BMMs (Fig. 4A-a), indicating the presence of uniform mesopores. First of all, the presence of the first inflection was observed under a relatively lower pressures (*P*/*P*_0_) of 0.20 < *P*/*P*_0_ < 0.40, which mainly arises from the spaces inside the small and narrow mesopores. In addition, the second inflection occurred at high relative pressures of 0.80 < *P*/*P*_0_ < 0.98, corresponding to a broader larger pore size distribution, which originated from the inter-particle aggregations.^28–30,47^

As shown in Fig. 4A (inset), their pore size distributions were calculated using the Barrett–Joyner–Halenda (BJH) method from the desorption branches of the isotherms, in which the shapes of all curves confirmed the presence of the bimodal mesopore structures with a narrow, small mesopore and broader, larger mesopore. As listed in Table 2, the small and large mean pore sizes of the pure BMMs were around 2.9 nm and 28.2 nm, respectively. In addition, the BET surface area (*S*_BET_) and pore volume (*P*_vol_) were 1205 m^2^ g^−1^ and 1.6 cm^3^ g^−1^ (Table 2, entry 1), respectively. Besides that, compared with that of the parent BMMs, *S*_BET_ and *P*_vol_ of ZnBMMs decreased to 942 m^2^ g^−1^ and 1.4 cm^3^ g^−1^, respectively. The bimodal mesopore sizes were also delinked to around 2.8 nm and 26.5 nm (Table 2, entry 2).

These results suggest that Zn was successfully located on the inner mesoporous surface of BMMs. Comparably, further decreases in *S*_BET_, *P*_vol_, and bimodal pore sizes were observed after immobilization with Z_1_, Z_2_, and Z_3_, respectively (Table 2, entries 3–5). Meanwhile, their positions of the inflection points in the N_2_ sorption isotherms shifted slightly to a lower relative pressure due to the mesoporous filling by the active species (Fig. 4A-b, -c, -d, and -e). These observations were based on the fact that the active species should be present inside the channels of the modified ZnBMMs.

Similarly, the N_2_ sorption isotherm of the MCM-41 (as shown in Fig. 4B-a) was type IV with a sharp step over a narrow range of relative pressure (0.20 < *P*/*P*_0_ < 0.35). Its *S*_BET_, *P*_vol_, and mean pore size were 710 m^2^ g^−1^, 0.7 cm^3^ g^−1^, 2.9 nm, respectively. Herein, the MCM-41-based structures had a rather well-defined pore size distribution with single and small mesopore channels that largely differs from the BMMs-based samples. After Zn-modification (Fig. 4B-b) and Z-immobilization (Fig. 4B-c, -d, and -e), the *S*_BET_, *P*_vol_, and mean pore size were found to decrease gradually (Table 2, entries 8–11), being consistent with the observations described previously (as shown in Fig. 4A).

As can be seen in Fig. 4C, the sorption isotherms of the SBA-15-based samples also revealed the typical type-IV isotherms with H1 type hysteresis. However, the observed mesopore filling step occurs in the range of *P*/*P*_0_ = 0.65–0.80, indicating the characteristic of the highly ordered 2D hexagonal mesostructure.^51,52^ Its mean mesopore size of 9.2 nm (Fig. 4C-a (inset)) with a narrow pore distribution was different from that of the BMMs- (around 2–3 nm) and MCM-41- (around 2–3 nm) based samples. The shapes of all isotherms almost remained intact after Zn-modification (Fig. 4C-b) and further Z-immobilization (Fig. 4C-c, -d, and -e). However, their corresponding *P*_vol_ and mean pore sizes decreased as compared to that of the parent SBA-15 (as shown in Table 2, entries 13–17).

These demonstrations were in good agreement with the estimates of the SAXS data (as shown in Fig. 1 and 2, as well as Table 1). As aforementioned, the correlations between the fractal dimension (*D*_m_ or *D*_s_) and their pore size distributions indicate that the *D*_m_ values of the BMMs-based samples increased, along with a greater densification tendency of the short worm-like mesopores channels. However, the *D*_s_ values of the MCM-41- and SBA-15-based samples decreased with the Zn-modifications and further Z-immobilizations. Therefore, we can speculate the reliability of the fractal dimensions and their maximum particle diameter deriving from SAXS analysis.

### 3.5 SEM/TEM images and EDX spectroscopy

**Fig. 5:**
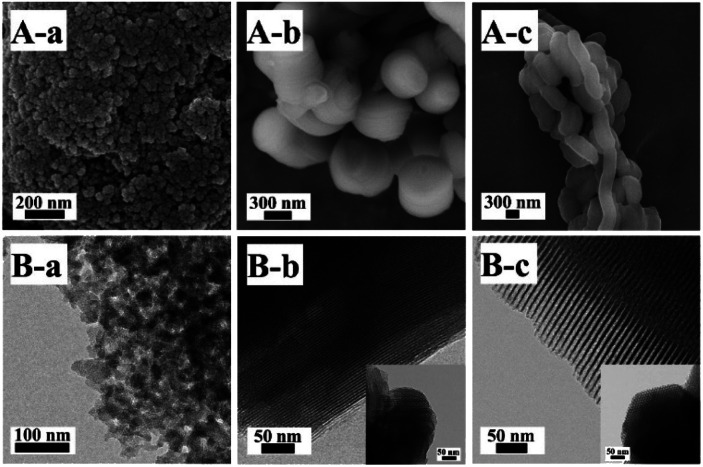
SEM (A) and TEM (B) images of (a) Z_2_ZnBMMs-100, (b) Z_2_ZnMCM-41-100, and (c) Z_2_ZnSBA-15-100. Inset shows a TEM image of the sample viewed along the hexagonal axis.

The SEM and TEM images of Z_2_ZnBMMs-100 are presented in Fig. 5A-a and B-a, which clearly show the appearances of the almost-spherical morphologies in the size of around 50 nm with disordered and uniform mesopores (around 3 nm), similar to that of the parent BMMs.^28–30,47^ However, Fig. 5A-b and B-b exhibit the cylindrical-like nanoparticles of Z_2_ZnMCM-41-100, a big difference from that of Z_2_ZnBMMs-100. In particular, the average particle size of Z_2_ZnMCM-41-100 was roughly estimated (considering 100 particles using Image J software) and found to be around 778 nm, as shown in Fig. S5 in the ESI section.† Meanwhile, the Z_2_ZnSBA-15-100 revealed the chain-like morphologies (Fig. 5A-c) with ordered and uniform mesopores in the size of around 8 nm (Fig. 5B-c).

On the basis of the SEM and TEM images, these estimated data further support the conclusions inferred from the fractal structure and the PDDF profiles (as shown in Fig. 1 and 2, as well as Table 1), especially for the globular morphologies of the BMMs-based samples with an approximately symmetric shape (as shown in Fig. 2, from -a to -e).

In contrast to the particle size, the effect of the pore size and shapes of the particles on the leaching process of active species was apparent, especially for the recycled catalysts. As shown in Table S1,† their loading of active species of the recycled catalysts (Z_2_ZnMCM-41-100 and Z_2_ZnSBA-15-100) were shown to be lower than that of Z_2_ZnBMMs-100. This suggested that Z_2_ZnBMMs-100 had short worm-like mesoporous channels, and was able to prevent active species loss in comparison with others, especially those pores around 2–3 nm that are close to the diameter of the molecular size. Meanwhile, from an enantioselectivity (ee) point of view (as shown in Table 1), the ee values of the former (28%, Table 1, entry 16) were larger than that of the latter (10 and 18%, entries 17 and 18 in Table 1). The long-range ordered structures of the MCM-41- and SBA-15-based samples with the relatively larger pore size might eventually lead to excess loss of the active species.

As shown in Fig. S2 in the ESI section,† the EDX elemental mappings of the Z_2_ZnSBA-15-100 catalyst further elucidated the existence of Zn and S apart from the dominant Si, O, and C elements. These phenomena indicate the homogeneous distributions of the above elements. Meanwhile, the presence of the S element mainly originated from the sulfonic acid groups in Z_2_.^32,47^

### 3.6 TGA profiles

**Fig. 6:**
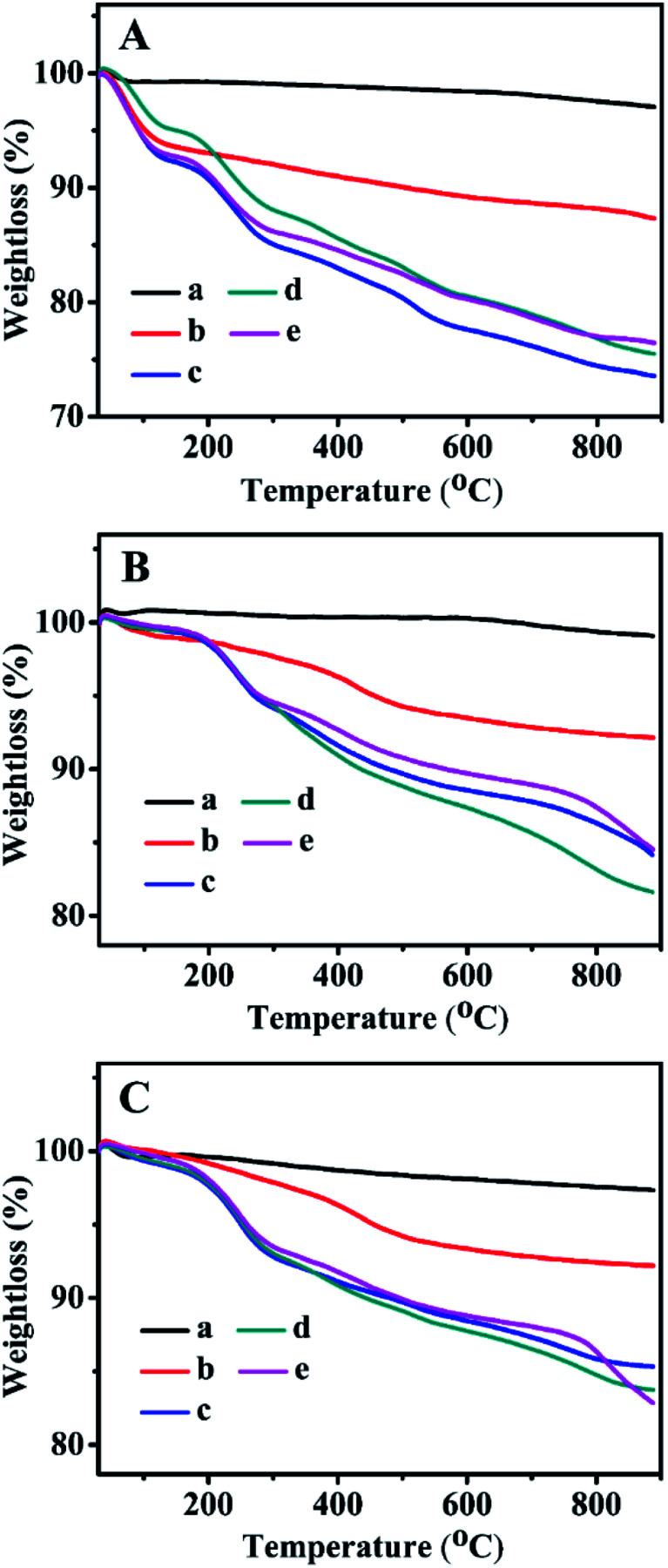
TGA curves of (A) BMMs-, (B) MCM-41-, and (C) SBA-15-based samples. (a) Pure mesoporous materials, (b) Zn-modified samples, (c) Z_1_-immobilized samples, (d) Z_2_-immobilized samples, and (e) Z_3_-immobilized samples, in which the molar ratios of Z/Zn for all samples were around 1 : 1.

The TGA curves of all related samples are presented in Fig. 6. As can be seen, their weight loss profiles from 30–200 °C were associated with the removal of the physically adsorbed and chemically adsorbed water inside the mesoporous channels, whereas the gradual weight loss of the Zn-grafted sample in the temperature range of 200–850 °C resulted from the decompositions of the zinc precursors containing organic functional groups.^29–31,47^ ZnBMMs exhibited a weight loss of about 5.77 wt% (Fig. 6A-b), while ZnMCM-41 and ZnSBA-15 underwent a slightly higher loss of about 6.43 wt% (Fig. 6B-b) and 6.87 wt% (Fig. 6C-b).

Additionally, the weight loss of the Z_1_, Z_2_, and Z_3_-immobilized samples in the range of 200–850 °C (as shown in Fig. 6A–C from -c to -e) was mainly caused by the decomposition of zinc precursors and transformation of organic compounds into a carbon-containing species, corresponding to the Z_1_, Z_2_, and Z_3_ percentage of about 12.51, 13.31, and 10.21 wt% in Z_1_-, Z_2_-, and Z_3_ZnBMMs (Fig. 6A-c, -d, and -e), respectively. Similarly, the weight loss of Z_1_-, Z_2_-, and Z_3_ZnMCM-41 (Fig. 6B-c, -d, and -e), and Z_1_-, Z_2_-, and Z_3_ZnSBA-15 (Fig. 6C-c, -d, and -e) were sequentially calculated to be 7.64, 10.87, 7.31; 6.46, 8.01, and 8.30 wt% in the temperature range of 200–850 °C.

In summary, the TGA results again indicated that the active species (Z) were successfully immobilized on the mesoporous surfaces, and these alterations give rise to the formations of the interfacial layer thickness, in good agreement with the demonstrations stemming from the SAXS patterns and other characterizations, such as the XRD patterns (Fig. 3) and N_2_ sorption isotherms (Fig. 4).

### 3.7 ICP-OES and elemental analysis

The elemental analysis results of N, C, and H in the as-prepared samples are summarized in Table S1 in the ESI section,† corresponding to the loading amount of Z_1_, Z_2_, and Z_3_ in fresh and recycled catalysts calculated on the basis of the N content. Meanwhile, the Zn content in each sample was analyzed by the ICP-OES method.

First, it can be seen that the contents of the N, C, and H elements increased after Z-immobilization, while the N content was virtually zero before and after Zn-grafting. On the basis of the N elemental data, Z-grafted amounts could be roughly estimated in Z_1_-, Z_2_-, and Z_3_ZnBMMs-100, or Z_1_-, Z_2_-, and Z_3_ZnMCM-41-100, or Z_1_-, Z_2_-, and Z_3_ZnSBA-15-100, corresponding to 10.11, 12.50, 9.49; 8.93, 11.83, 8.86; and 7.49, 7.43, and 8.74 wt%, respectively. Additionally, the Zn contents presented in Table S1† almost remained the same as around 5.77–7.30 wt%. In particular, comparison experiments were performed to elucidate whether Zn may play a role as a critical bridge among the catalysts and mesoporous silicas,^29^ as shown in Table S2 in the ESI section.† Moreover, these results indicated that the immobilization of Z_2_ on BMMs by coordination bonding (Table S2, entry 2†) was more stable than that of hydrogen bonding without Zn (Table S2, entry 1†), and further confirmed that Z_1_, Z_2_, and Z_3_ were successfully immobilized on the mesoporous surfaces.

### 3.8 FT-IR and UV-Vis spectra

**Fig. 7:**
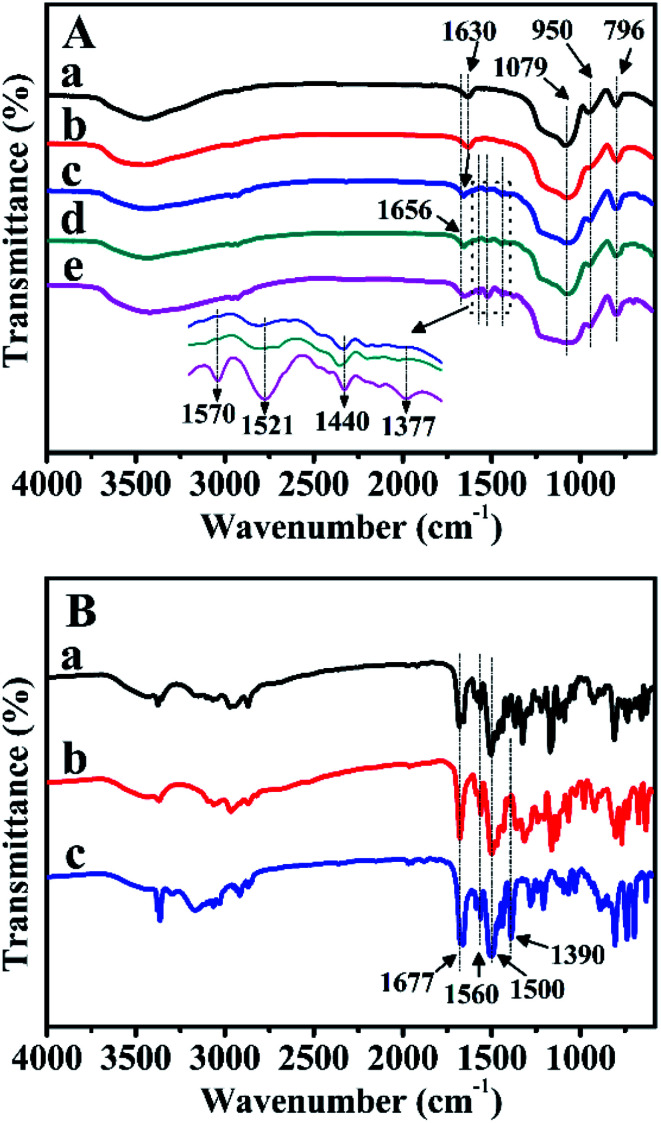
FT-IR spectra of (A) BMMs-based samples. (a) Pure mesoporous materials, (b) Zn-modified samples, (c) Z_1_-immobilized samples, (d) Z_2_-immobilized samples, and (e) Z_3_-immobilized samples, in which the molar ratios of Z/Zn for all samples were around 1 : 1. (B-a) Z_1_, (B-b) Z_2_, and (B-c) Z_3_.

FT-IR spectra were further carried out to verify the characteristic functional groups of the prepared samples after Zn-modification and Z-immobilization. As illustrated in Fig. 7A-a, for pure BMMs, the bands observed at 1630, 1079, 950, and 796 cm^−1^ were ascribed to the vibrational features of H–O–H, Si–O–Si, Si–OH, and Si–O, respectively. Comparably, for ZnBMMs (Fig. 7A-b), the weak band centered at 950 cm^−1^ could be explained by the formation of the Si–O–Zn structure.^29–31^

Especially, for Z_1_, Z_2_, and Z_3_, Fig. 7B indicates that the main bands located at 1677, and 1500 cm^−1^ could be attributed to the C=O and C=N stretching vibrations in the amide functional group,^29,30^ respectively, which should originate from the pyridine rings. Significantly, as shown in Fig. 7A-c, -d, and -e, after Z_1_-, Z_2_-, and Z_3_-immobilization, several additional bands appeared in the region of 1660–1370 cm^−1^. The band at 1630 cm^−1^ belonging to the C=O vibration was slightly shifted to higher wavenumbers (1656 cm^−1^), as compared with that of either pure BMMs or Zn-grafted (Fig. 7A-a and -b), indicating that the Z_1_, Z_2_, and Z_3_ were incorporated into the BMMs surfaces. Moreover, the features band (C=N) at 1500 cm^−1^ in the pyridine rings shifted to 1440 cm^−1^. This is likely due to the electron transfer from N of the pyridine rings to Zn^2+^ for the coordination.^29–31^ The shiftiness of the features band for the C=N stretching vibrations again proved the successful Z-immobilization. Similar phenomena appeared using MCM-41 and SBA-15 as supports, as shown in Fig. S3-A and -B (from -a to -e).†

In summary, these observations further demonstrate that the Z_1_, Z_2_, and Z_3_ were successfully immobilized on the Zn-grafted surface *via* coordination interaction. This is a big difference from that of physical mixtures between the mesoporous support and the Zn or Z species.

**Fig. 8:**
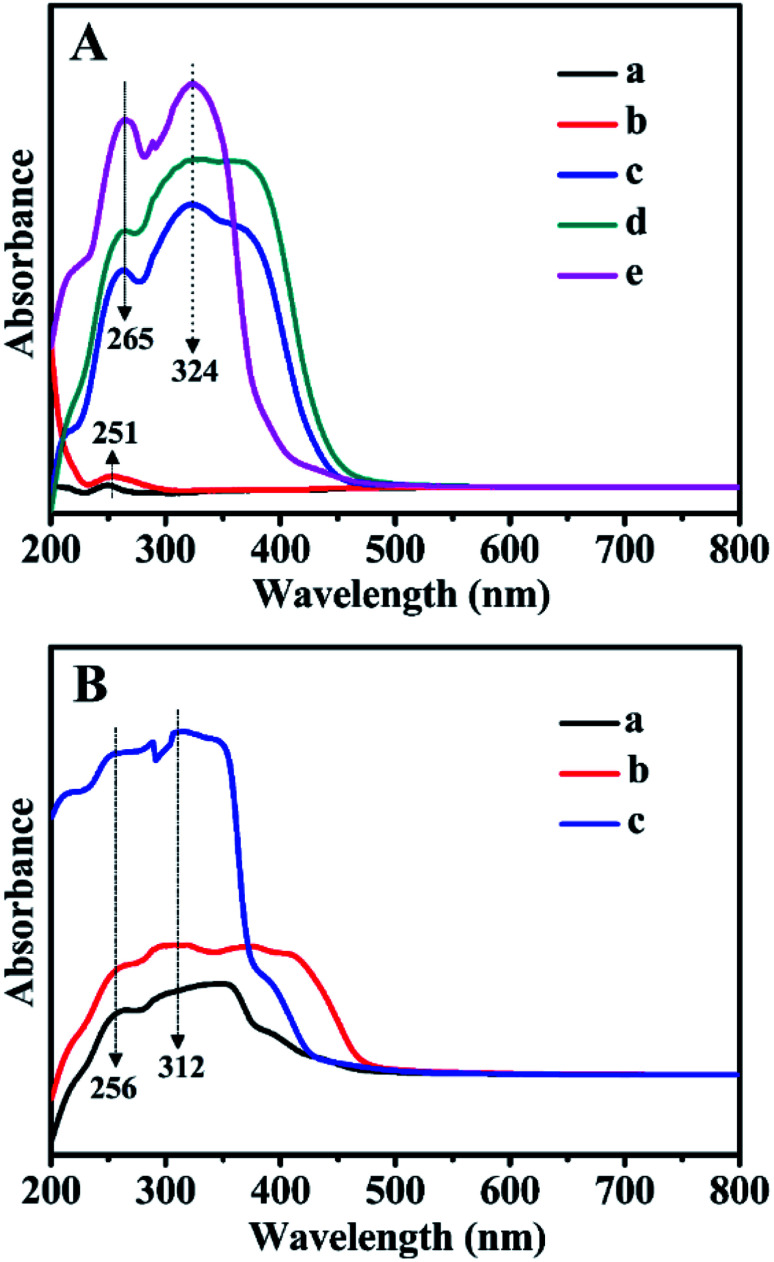
UV-Vis DR spectra of (A) BMMs-based samples. (a) Pure mesoporous materials, (b) Zn-modified samples, (c) Z_1_-immobilized samples, (d) Z_2_-immobilized samples, and (e) Z_3_-immobilized samples, in which the molar ratios of Z/Zn for all samples were around 1 : 1. (B-a) Z_1_, (B-b) Z_2_, and (B-c) Z_3_.

Fig. 8 presents the absorbance UV-Vis spectra of all related samples in the wavelength range of 200–800 nm. As can be seen in Fig. 8B, for Z_1_, Z_2_, and Z_3_, the absorption bands observed at 256 and 312 nm were related to the π → π* and n → π* transitions of the C=C and C=O bonds in the bipyridyl structures. Comparably, for BMMs (Fig. 8A-a) or ZnBMMs (Fig. 8A-b), an extremely weak absorption peak at around 251 nm stemmed from the nanoporous effect of pure BMMs. As shown in Fig. 8A-c, -d, and -e, several additional absorption peaks of Z-immobilized matrices that appeared in the range of 210–380 nm could be attributed to the bipyridyl structures of Z_1_, Z_2_, or Z_3_. Meanwhile, the occurrences of the characteristic peaks red-shifted from 256 to 265 nm and 312 to 324 nm. Fig. S4-A and -B† present nearly identical spectra as that shown in Fig. 8A.

Based on the FT-IR and UV-Vis DR spectra, these observations again indicate the successful immobilizations of the active species (Z) onto the Zn-grafted mesoporous surfaces, resulting in the formation of Si–O–Zn or Si–O–Zn–Z structures *via* coordination and covalent bond. These phenomena brought further indirect evidence that the fractal structures and interfacial layers of these heterogeneous catalysts could be achieved.

### 3.9 Characterization of the recycled catalysts

The SAXS method was also employed to characterize the fractal features of the 3^rd^ recycled catalysts (Z_2_ZnBMMs-100, Z_2_ZnMCM-41-100, and Z_2_ZnSBA-15-100), and the detailed results are shown in Table 1 (entries 16–18), as well as Fig. 1 and 2. As can be seen in Table 1 (entry 4), the *D*_m_ values and the interface layer thickness of the 3^rd^ recycled Z_2_ZnBMMs-100 were larger than that of the fresh catalyst. Its PDDF profile (Fig. 2B-f) was more asymmetric than that of the fresh catalyst (Fig. 2B-d). Moreover, its maximum particle size was about 66 nm (Fig. 2B-f), which is larger than that of the fresh catalyst (Fig. 2B-d).

Meanwhile, the *D*_s_ values of the 3^rd^ recycled Z_2_ZnMCM-41-100 (Table 1, entry 17) and Z_2_ZnSBA-15-100 (Table 1, entry 18) were larger than that of the fresh catalysts (Table 1, entries 9 and 14), respectively. Their ln[*q*^4^*I*(*q*)] ∼ *q*^2^ curves (Fig. S1-Q and -R†) belonged to the positive deviation, which is nearly identical to that of the fresh catalyst (Fig. S1-I, and -N†).

Their XRD patterns, as shown in Fig. 3A, B and C-f, indicated that their relative peak intensities at 2*θ* of about 1° or 2° became weaker than that of the corresponding fresh catalysts (Fig. 3A, B and C-d), suggesting that the order degree of the uniform mesopore structure declined. Additionally, their N_2_ adsorption–desorption isotherms (as shown in Fig. 4A, B and C-f) and the corresponding textural parameters (as summarized in Table 2, entries 6, 12, and 18) demonstrated that both *S*_BET_ and *P*_vol_ values of the 3^rd^ recycled catalysts were lower than that of the fresh catalysts. These abovementioned results may be related to the carbon depositions originating from the accumulation of organic products in the aldol reaction or certain damages of the mesoporous structures. Moreover, their elemental analysis and ICP-OES results, as shown in Table S1,† indicated that the loading of the active species and Zn of the 3^rd^ recycled catalysts were both lower than that of the fresh catalysts.

### 3.10 TON and TOF results

For comparison, the turnover number (TON) and turnover frequency (TOF) values under the catalysis of Z_1_-, Z_2_-, and Z_3_ZnBMMs-100 in this work are provided in Table S3 in the ESI section.† In particular, the maximum TON and TOF values of the catalytic aldol reaction using *p*-nitrobenzaldehyde as a substrate were calculated to be 4.85 and 1.54 [(g h)^−1^] under the catalysis of Z_1_ZnBMMs-100 (Table S3, entry 1a†).

In addition, the comparative experiments showed extremely poor performance of the catalytic aldol reaction using *p*-methoxybenzaldehyde with the electron-donating substituent (as shown in Fig. S6 in the ESI section†).

## 4. Conclusions

In conclusion, a series of heterogeneous catalysts (Z_1_(or Z_2_, or Z_3_)ZnBMMs-100, Z_1_(or Z_2_, or Z_3_)ZnMCM-41-100, and Z_1_(or Z_2_, or Z_3_)ZnSBA-15-100) with various active species (Z) were successfully prepared, and their catalytic performances for asymmetric aldol reactions were preliminarily evaluated. Meanwhile, their structural features and physicochemical properties were demonstrated thoroughly by SAXS patterns and other characterizations. The possible relationships between the fractal structures of these heterogeneous catalysts and their catalytic performance (including catalytic activity and stereoselectivity) were investigated. In particular, the fractal evolution of the Z-immobilized ZnBMMs, ZnMCM-41, and ZnSBA-15 before and after modification suggested the successful Z-introductions onto the inner-mesoporous channels, which was further corroborated by XRD patterns and N_2_-sorption isotherms. Moreover, the influences of the mesoporous fractal structures of the three types of silica-based catalysts and the molecular volumes of aldehydes on the catalytic behaviors (yield, dr and ee) of the asymmetric aldol reactions were obvious. Overall, these results elucidated the reliability of the SAXS technique in revealing the fractal structure and their catalytic performance relationships.

## Author contributions

Guangpeng Xu and Liujie Bing: investigation, writing – original draft preparation. Bingying Jia: data curation. Shiyang Bai: formal analysis, validation. Jihong Sun: supervision, conceptualization, methodology.

## Conflicts of interest

The authors declare no conflict of interest.

## Supplementary Material

RA-012-D2RA00971D-s001
