# Supplementary material for: Comparison of mesoporous fractal characteristics of silica-supported organocatalysts derived from bipyridine-proline and resultant effects on the catalytic asymmetric aldol performances
Source: RSC Adv. 2022 Apr 7;12(17):10800–14. doi: 10.1039/d2ra00971d (PMC8988269; doi:10.1039/d2ra00971d)
Supplement: RA-012-D2RA00971D-s001 [file RA-012-D2RA00971D-s001.pdf]

Electronic Supplementary Material (ESI) for RSC Advances.
This journal is © The Royal Society of Chemistry 2022

Electronic Supporting Information Summary

Comparison of mesoporous fractal characteristics of silica-supported organocatalysts derived from bipyridine-proline and resultant effects on catalytic asymmetric aldol performances

Guangpeng Xu,[a] Liujie Bing,[a] Bingying Jia,[a] Shiyang Bai [a] and Jihong Sun *[a]

[a]*Beijing Key Laboratory for Green Catalysis and Separation, Department of Environmental and Chemical Engineering, Beijing University of Technology, Beijing 100124, China*

Corresponding author: jhsun@bjut.edu.cn

1. Experimental section

1.1. Materials

Ionic cetyltrimethylammonium bromide (CTAB, 99% of purity) was purchased from Sinopharm Chemical Reagent Beijing Co., Ltd. Tetraethyl orthosilicate (TEOS, 98%) was purchased from Bailingwei Technology Beijing Co., Ltd. Non-ionic surfactant Pluronic tri-block copolymer (P123, Mn: 5800) was supplied by Sigma-Aldrich company. Zinc acetate dihydrate (99%), *p*-nitrobenzaldehyde (99.8%), 2-naphthaldehyde (99.8%), 9-anthracenecarboxaldehyde (99.8%), and 1-pyrenecarboxaldehyde (99.8%) were supplied by Shanghai Aladdin Biochemical Technology Co., Ltd. Dimethyl sulfoxide (DMSO, 99.9%), petroleum ether (60-90 ^{o}C), ethyl acetate (EtOAc, 99.9%), dichloromethane (CH_2Cl_2, 99.9%), trifluoroacetic acid (TFA, 99.9%), and cyclohexanone (99.9%) were provided by Tianjin Fuchen chemical reagents factory. Anhydrous methanol (99.9%) and ethanol (99.9%), anhydrous Na_2SO_4 (99.5%), and ammonium hydroxide (25%, $NH_3{\cdot}H_2O$) were obtained from Beijing chemical works. 0.25 mm SDS silica gel coated glass plates (60F254) and silica gel (200-300 mesh) was purchased from Qingdao Haiyang Chemical Co., Ltd. All the chemicals were purchased as reagent grade and used without further purification. All of the solvents and reagents were of A.R. grade. Deionized water was used in all experiments.

1.2. Characterizations

The SAXS experiments were carried out using synchrotron radiation at the 1W2A X-ray beamline at Beijing Synchrotron Radiation Facility (BSRF). The wavelength of the incident X-ray was 0.154 nm. The sample-to-detector distance for SAXS was 1.59 m, calibrated with the diffraction ring of a standard sample. The scattering vector

magnitude q ranged from 0.08 to 3.05 nm^{-1} for the experiment reported in this paper. The intensity of scattering, $I(q)$ was measured as a function of q, where q was considered as $4\pi\sin\theta/\lambda$. The sample was loaded into a sample cell and sealed with scotch tape on a groove. The thickness of the sample cell was approximately 1 mm. The scattering image was collected with an exposure time of 5 min by the single-frame mode with a 'multi-read' of 2 times. The two-dimensional SAXS images were transferred to one-dimensional data by using the Fit2D software (http://www.esrf.eu/computing/scientific/FIT2D),[1] and further processed with the *S* program package.[2] In this study, a slit-collimated beam was used, for which the mass fractals (D_m) or surface fractals (D_s) were calculated according to the power law. Besides, the powder XRD patterns were recorded on a D6 Advance X-ray diffractometer (Beijing Pu Analysis General Instrument Co., Ltd, Beijing, China) using Cu-Kα radiation (λ = 0.154056 nm, 36 kV, 20 mA) at a scanning speed of 1 ^{o} and 4 ^{o} per min in the 1 - 10 ^{o} and 10 - 50 ^{o} 2θ range for the experiments, respectively. The textural properties of related samples were determined from N_2 sorption isotherms measured at 77 K using JWGB JW-BK300 analyzer. Prior to each adsorption measurement the samples were outgassed under vacuum at 353 K for at least 5 h. The specific surface area was calculated using the Brunauer-Emmett-Teller (BET) method and the pore size distribution was calculated from the isotherm using the Barrett-Joyner-Halenda (BJH) model. Besides that, the total pore volume was determined from the N_2 adsorbed amount at a relative vapor pressure (P/P_0) of approximately 0.99. The SEM images were performed on a Hitachi field-emission scanning electron microscope (S-4800) with an acceleration voltage of 15.0 kV. The morphology and elemental composition of the materials were acquired with a TEM microphotograph (JEOL, JEM-2100F, Japan) coupled with EDX spectroscopy. The TGA was carried out on a PerkinElmer Simultaneous Thermal Analyzer (STA-8000) instrument from 30 to 900 ^{o}C at a heating rate of 10 ^{o}C/min under the N_2 atmosphere with a flow rate of 20 mL/min. The content of nitrogen was measured by vario MICRO cube elemental analyzer and the metal Zn concentrations were determined by ICP-OES using Optima 8300, PerkinElmer. The FT-IR spectra were measured recorded on a Bruker Tensor 2 spectrometer (Bruker Optik GmbH, Germany) via the KBr tablet method, in which the spectral resolution was 4 cm^{-1}, and 32 scans were recorded for each spectrum. The UV-vis DR spectra were carried out in the wavelength range of 200-800 nm with a Shimadzu UV-2600 spectrophotometer (Shimadzu, Japan). The *dr* and *ee* values were determined by HPLC analyses on a Agilent Technologies 1200 system equipped with a photodiode array detector, using Chiralpak AD-H column (25 cm × 0.46 cm) by Daicel Chemical Ind., Ltd. Besides that, the chemical identity of representative chiral ligands (Z_1, Z_2, and Z_3) and all aldol products has been confirmed by nuclear magnetic resonance (^{1}H- or ^{13}C-NMR) in our previous report.[3]

2. Additional results

2.1. Porod plots

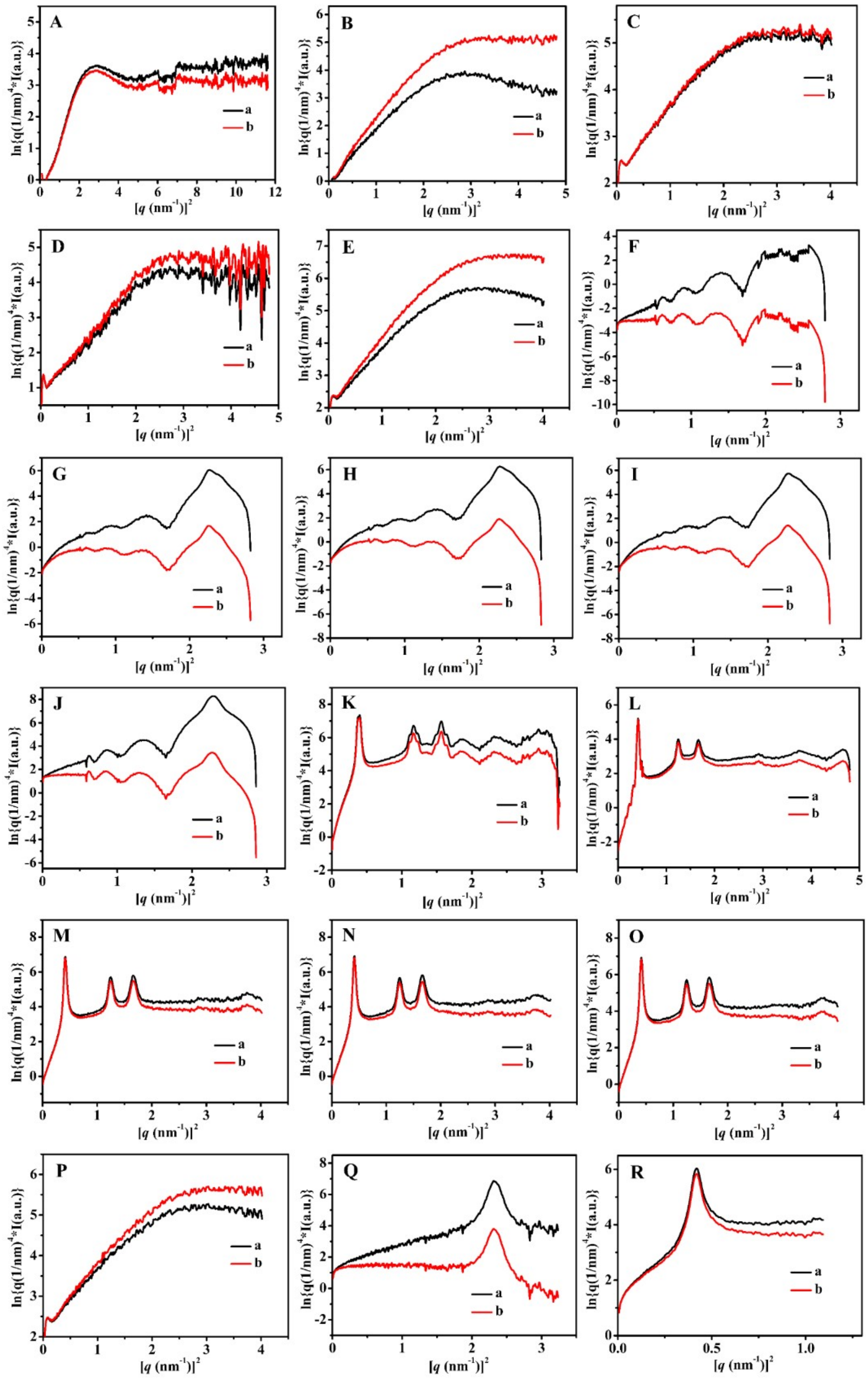

Fig. S1. *$ln[q^4I(q)] \sim q^2$* curves of (A) BMMs, (B) ZnBMMs, (C) Z_1ZnBMMs-100, (D) Z_2ZnBMMs-100, (E) Z_3ZnBMMs-100, (F) MCM-41, (G) ZnMCM-41, (H) Z_1ZnMCM-41-100, (I) Z_2ZnMCM-41-100, (J) Z_3ZnMCM-41-100, (K) SBA-15, (L) ZnSBA-15, (M) Z_1ZnSBA-15-100, (N) Z_2ZnSBA-15-100, (O) Z_3ZnSBA-15-100, and 3rd recycled (P) Z_2ZnBMMs-100, (Q) Z_2ZnMCM-41-100, (R) Z_2ZnSBA-15-100, in which, (a) Porod plot data with deviation, (b) deviation corrected Porod plot data.

2.2. EDX spectra

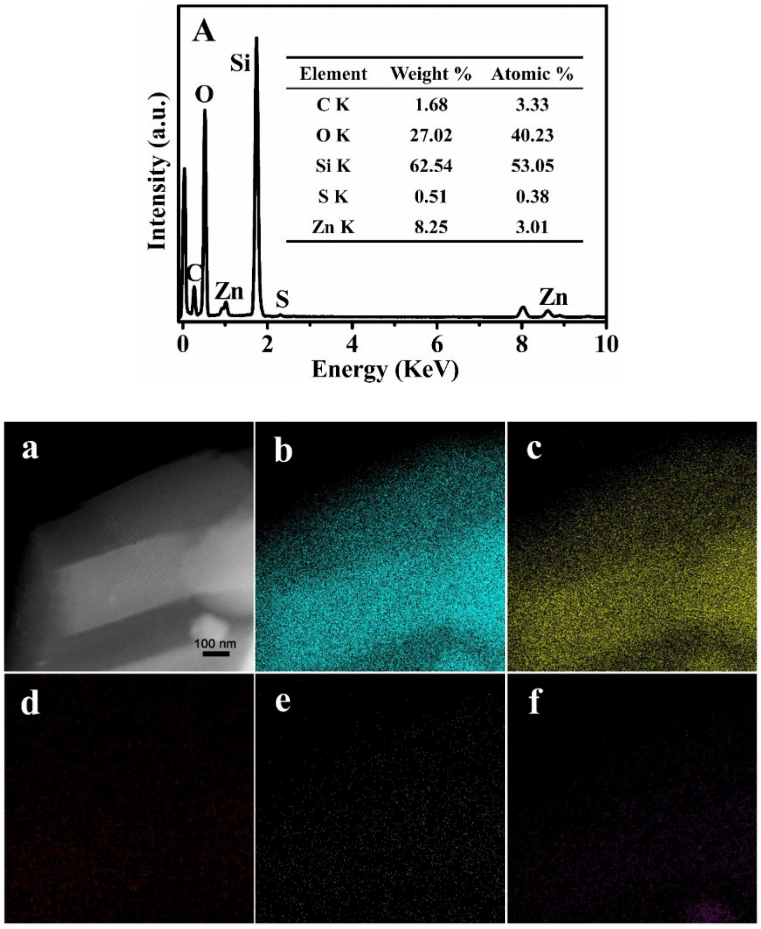

Fig. S2. (A) EDX spectra and results of Z_2ZnSBA-15-100; (a) TEM image and its corresponding EDX mapping spectra of (b) Si, (c) O, (d) C, (e) S, (f) Zn.

2.3. ICP-OES and Elemental analysis

Table S1. Collection of elemental composition and Zn concentrations of all related samples.

Samples[a]	N content wt%[b]	C content wt%	H content wt%	Loading of Z wt%[b]	Loading of Z wt% (TGA results)[c]	Loading of Zn wt% (ICP results)[d]
BMMs	-	0.45	1.07	-	-	-
ZnBMMs	-	2.20	2.05	-	-	6.14
Z_1ZnBMMs-100	1.62	7.36	1.97	10.11	12.51	6.82
Z_2ZnBMMs-100	1.85	10.34	2.25	12.50	13.31	6.64
Z_3ZnBMMs-100	1.66	9.96	2.41	9.49	10.21	7.30
Z_2ZnBMMs-100[e]	0.58	6.12	1.41	3.92	--	3.10
MCM-41	-	0.28	1.29	--	--	--
ZnMCM-41	-	1.04	1.91	--	--	6.52
Z_1ZnMCM-41-100	1.43	6.61	1.62	8.93	7.64	6.68
Z_2ZnMCM-41-100	1.75	8.91	2.19	11.83	10.87	6.96
Z_3ZnMCM-41-100	1.55	7.35	2.05	8.86	7.31	7.02
Z_2ZnMCM-41-100[e]	0.29	2.45	0.90	1.96	--	3.38
SBA-15	-	0.27	1.81	-	-	-
ZnSBA-15	-	1.40	1.59	-	-	6.73
Z_1ZnSBA-15-100	1.20	7.10	2.34	7.49	6.46	5.77
Z_2ZnSBA-15-100	1.10	6.98	1.71	7.43	8.01	6.28
Z_3ZnSBA-15-100	1.53	9.33	2.19	8.74	8.30	6.69
Z_2ZnSBA-15-100[e]	0.16	2.65	0.92	1.08	--	3.33

[a] The molar ratios of Z/Zn for all samples were around 1:1. [b] The values of the loading of Z were calculated by the nitrogen content. [c] Determined by TGA results. [d] Determined by ICP-OES. [e] The recycled catalyst after three runs.

2.4. FT-IR and UV-Vis DR Spectra

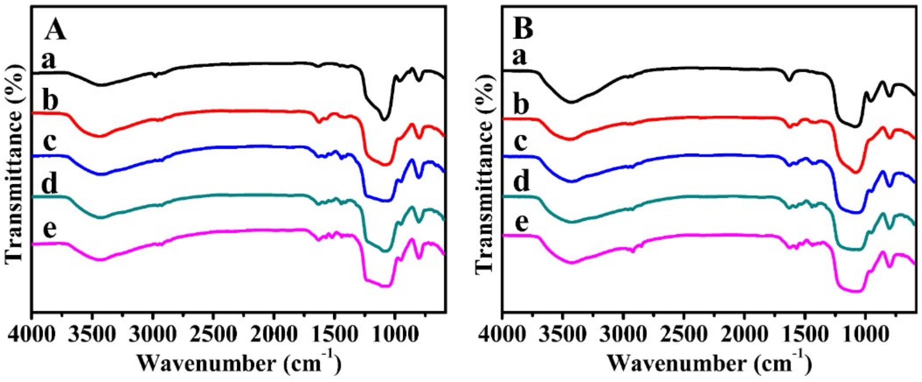

Fig. S3. FT-IR spectra of (A) MCM-41-, and (B) SBA-15-based samples. (a) pure mesoporous materials, (b) Zn-modified samples, (c) Z_1-immobilized samples, (d) Z_2-immobilized samples, and (e) Z_3-immobilized samples, in which, the molar ratios of Z/Zn for all samples were around 1:1.

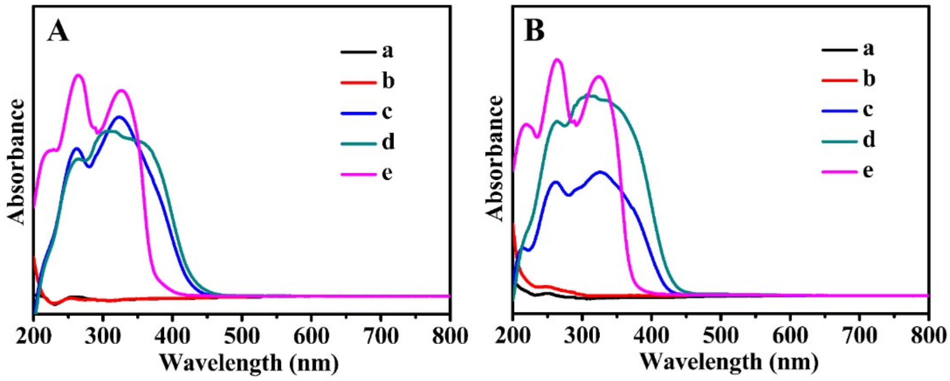

Fig. S4. UV-vis DR spectra (A) MCM-41-, and (B) SBA-15-based samples. (a) pure mesoporous materials, (b) Zn-modified samples, (c) Z_1-immobilized samples, (d) Z_2-immobilized samples, and (e) Z_3-immobilized samples, in which, the molar ratios of Z/Zn for all samples were around 1:1.

2.5. Particle size distribution of Z_2ZnMCM-41-100

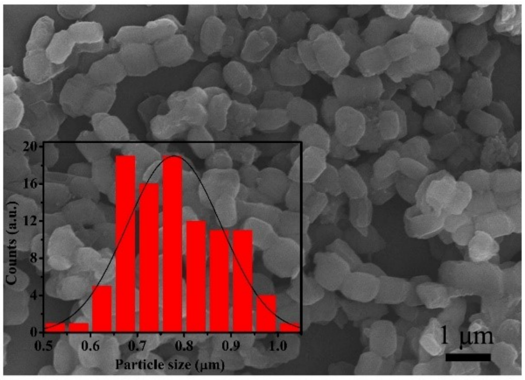

Fig. S5. SEM image and particle size distribution (inset) of Z_2ZnMCM-41-100.

2.6. The comparison between current research and literature

Table S2. Comparison of catalytic activity (yield) according to the reported hybrid catalysts (Z_0-BMMs) on the asymmetric aldol reaction of *p*-nitrobenzaldehyde with cyclohexanone, as well as leaching of active species.

Entry	Catalyst	Yield (%)	N content wt% [a]	Loading of Z wt% [a]	*reference*
1	Z_2-BMMs [b]	91	0.82	5.54	This work
2	Z_2ZnBMMs	97	1.85	12.50	This work
3	Z_0-BMMs-15 [c]	80	2.55	11.52	4
4	Z_2ZnBMMs [d]	85	0.58	3.92	This work
5	Z_0-BMMs-15 [e]	10	--	--	4

[a] The values of the loading of Z were calculated by the nitrogen content. [b] Z_2 grafted BMMs was prepared without Zn by post treatment in this work. [c] Z_0: (2*S*, 2′S)-*N*,*N*′-([2,2′-bipyridine]-3,3′-diyl)bis (pyrrolidine-2-carboxamide).[4]

[d] The catalyst was reused for a third run. [e] The catalyst was reused for a second run.

Based on the experimental results of this study and our group previous literature data,[4] major advantages for these heterogeneous catalysts (Z_1-, or Z_2-, or Z_3ZnBMMs, Z_1-, or Z_2-, or Z_3ZnMCM-41, and Z_1-, or Z_2-, or Z_3ZnSBA-15) by comparison with that of the hybrid catalyst (Z_0-BMMs), which were prepared by Tang *et al.* via grafting Z_0 onto the surface of bimodal mesoporous silicas (BMMs). Taking Z_2ZnBMMs as an example, as shown in Table S2.

In particular, for the purpose of comparison, Z_2 grafted BMMs was prepared without Zn by post treatment in this work. As can be seen in Table S2, from the perspective of their catalytic activity, Z_2ZnBMMs as catalyst showed an excellent reusability with high activity (yield of 85%, Table S2, entries 4), however, Z-BMMs, if reused more than 2 times, showed rapid catalytic deactivation (yield from 80 to 10%, Table S2, entries 3 and 5).

In addition, on the basis of the N elemental data, Z_2-grafted amounts could be roughly estimated in Z_2-BMMs and Z_2ZnBMMs, corresponding to 5.54 and 12.50 wt% (Table S2, entries 1 and 2), respectively. Obviously, further confirming that the Z_2 were successfully immobilized on the mesoporous surfaces.

In summary, we found that the immobilization of Z_2 on BMMs by coordination bonding (Table S2, entries 2) was more stable than that of hydrogen bonding without Zn (Table S2, entries 1).

2.7. TON and TOF results

Table S3. Comparison of TON and TOF under the catalysis of Z_1-, Z_2-, Z_3ZnBMMs-100.

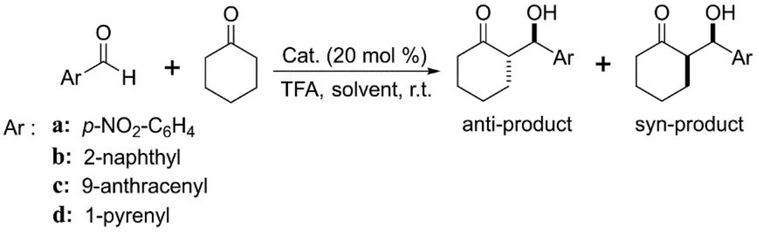

Entry	Catalyst	Reaction	TON	TOF [(g · h)$^{-1}$]
1	Z_1ZnBMMs-100	a	4.85	1.54
		b	3.80	1.21
		c	0.95	0.30
		d	2.00	0.64
2	Z_2ZnBMMs-100	a	4.85	1.42
		b	4.20	1.23
		c	1.15	0.34
		d	1.90	0.56
3	Z_3ZnBMMs-100	a	2.85	0.82
		b	1.00	0.29
		c	0.55	0.16
		d	0.50	0.14

$$TOF = \frac{n_1}{m_1 \times n_2 \times t} \quad (1)$$

$$TON = \frac{n_1}{n_2} \quad (2)$$

Where n_1 denote the moles of the product molecules, n_2 denote the moles of the involved in catalysis Z, m_1 denote the weight of the used catalyst, and t denotes the whole catalytic reaction time, respectively. The turnover frequency (TOF) and turnover number (TON) can be described in detail as Equations 1 and 2.

As shown in Table S3, taking Z_1-, Z_2-, Z_3ZnBMMs-100 as an example, comparison of TON and TOF under above catalytic systems. The TOF values presented in Table S3 almost remained the same as around 0.14 - 1.54 $[(g{\cdot}h)^{-1}]$, and the TON values in the range of 0.50 - 4.85.

2.8. Comparative experiments for asymmetric aldol reaction

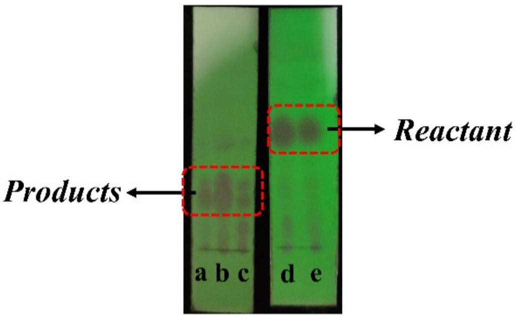

Fig. S6. Comparison of the results of TLC separation on the asymmetric aldol reaction between the different aldehyde and cyclohexanone in the presence of (a) Z_1ZnBMMs-100, (b) Z_2ZnBMMs-100, (c) Z_3ZnBMMs-100, (d) Z_2, (e) Z_2ZnBMMs-100, in which, *p*-nitrobenzaldehyde as a reactant for (a), (b), and (c), respectively; and *p*-methoxybenzaldehyde as a reactant for (d) and (e), respectively.

In addition, the aldol reaction with electron-donating substituent *p*-methoxybenzaldehyde as a reactant by comparison with *p*-nitrobenzaldehyde were carried out, but the results were not good presumably due to the inappropriate catalyst and substrate. Unfortunately, the bands of aldol products were almost not reflected on TLC plates, as shown in Fig. S6.

3. References

1 A. P. Hammersley, *J. Appl. Crystallogr.*, 2016, **49**, 646-652.
2 Z. Li, *Chin. Phys. C.*, 2013, **37**, 110-115.
3 G. Xu, Y. Zhang, J. Sun, S. Bai and H. Zhao, *ChemistrySelect*, 2020, **5**, 10996-11003.
4 Z. Tang, J. Sun, H. Zhao, S. Bai, X. Wu and H. Panezai, *Microporous Mesoporous Mater.*, 2018, **260**, 245-252.
